# Harnessing genetic, chemical, and transcriptomic analyses guided the selection of *Cistus creticus* subsp. *creticus* genotypes rich in labdane-type diterpenes

**DOI:** 10.1007/s00425-026-05072-1

**Published:** 2026-07-15

**Authors:** Stefanos Kostas, Symela Ntoanidou, Christos Bazakos, Eirini Sarrou, Nausica Rafailidou, Antigoni Papanikolaou, Antonios Makris, Aikaterini-Angeliki Kotoula, Efstathios Hatziloukas, Athanasios Economou, Angelos K. Kanellis

**Affiliations:** 1 Laboratory of Floriculture, Department of Horticulture, Aristotle University of Thessaloniki, 54124 Thessaloniki, Greece; 2 Group of Biotechnology of Pharmaceutical Plants, Laboratory of Pharmacognosy, Department of Pharmaceutical Sciences, Aristotle University of Thessaloniki, 54124 Thessaloniki, Greece; 3 Institute of Plant Breeding and Genetic Resources, ELGO-DIMITRA, Thessaloniki-Thermi, Greece; 4 Department of Comparative Development and Genetics, Max Planck Institute for Plant Breeding Research, Carl-Von-Linne-Weg 10, 50829 Cologne, Germany; 5 Institute of Applied Biosciences, Centre for Research and Technology Hellas (INAB-CERTH), P.O. Box 60361, Thermi, 57001 Thessaloniki, Greece; 6 Laboratory of Molecular Biology and Genetics, Department of Biological Applications and Technologies, University of Ioannina, 45110 Ioannina, Greece

**Keywords:** Ladano, Molecular markers, ISSR, GC–MS, Gene expression

## Abstract

**Main conclusion:**

**The present study connects the genetic diversity of** ***Cistus creticus*** **to its chemical profile, identifying elite genotypes and key biosynthetic genes for enhancing the production of valuable labdane diterpenes.**

**Abstract:**

*Cistus creticus*, a Mediterranean shrub, is known for its resin ladano, which is rich in pharmacologically active labdane-related diterpenes (LRDs). However, the genetic and molecular basis for the LRDs content variation among natural populations remains uncharacterized. This study aims to fulfill this lacuna by investigating a potential link between genetic diversity and chemotypic profile across 91 genotypes from seven different populations across Greece and identifying elite germplasm by uncovering the molecular and regulatory mechanisms underlying LRDs biosynthesis. The genetic analysis, employing ISSR markers, revealed significant genetic differentiation among populations (Φ_ST_ = 0.294) that mirrored distinct chemotypic profiles. Genotypes clustered by origin with Chalkidiki and Sises being the most homogenous, high-yielding, and superior in LRDs production. The transcriptomic analysis of low (C1) vs. high (C18) LRDs genotypes identified 24,489 up-regulated genes, including key diterpene synthases [labda-7,13-dien-15-ol synthase (*LDDS2*), *ent*-copalyl diphosphate synthase, copal-8-ol diphosphate synthase (*CLS*)] and numerous transcription factors among which WRKY, bHLH and bZIP were strongly correlated with LRD accumulation. This work provides an initial representation of the regulatory landscape by identifying elite genotypes, key biosynthetic genes, and transcription factors linked to specific LRDs for promising breeding programs, future targeted experiments, and metabolic engineering.

**Graphical abstract:**

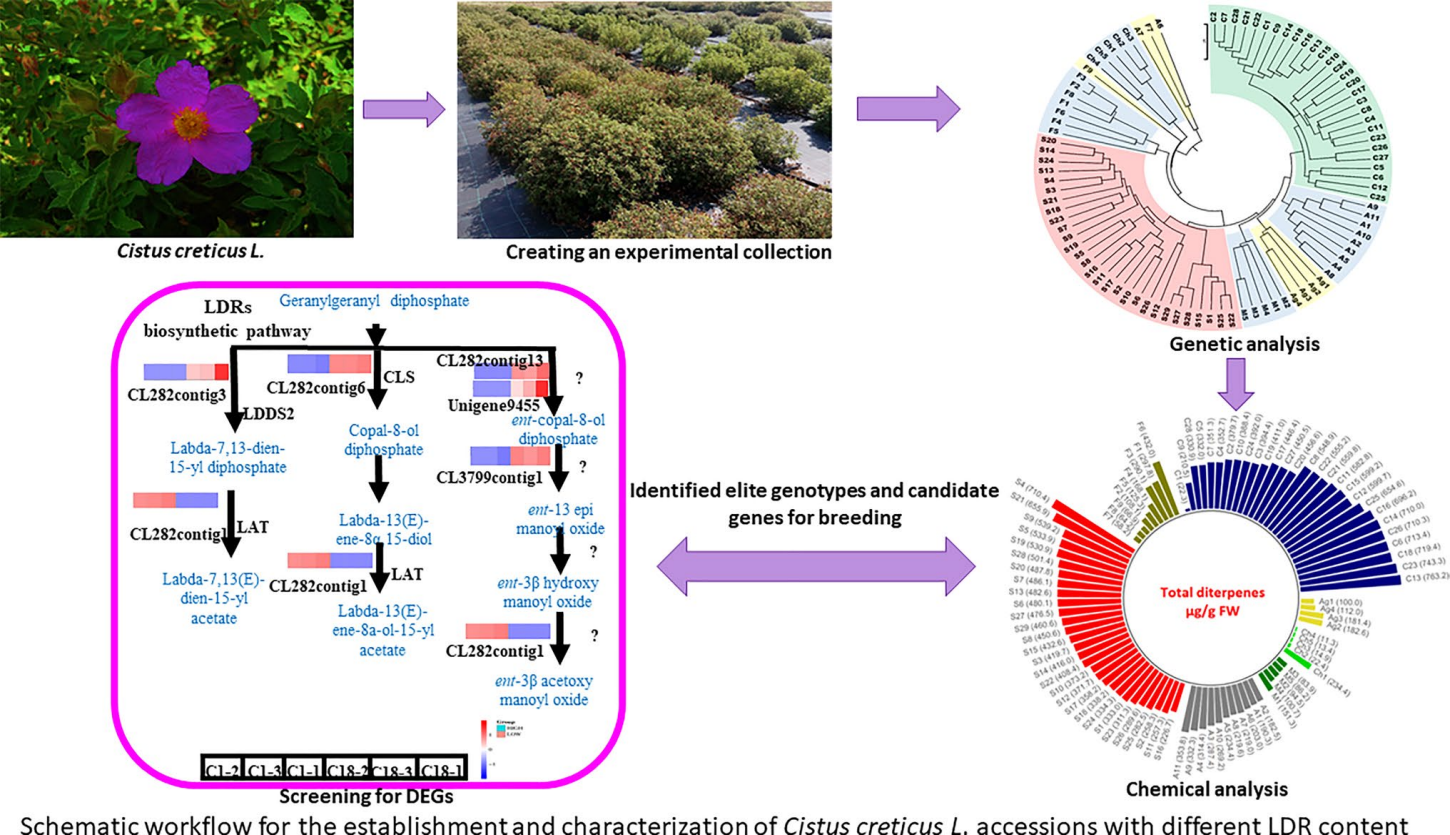

**Supplementary Information:**

The online version contains supplementary material available at 10.1007/s00425-026-05072-1.

## Introduction

*Cistus creticus* L., commonly known as rock rose, is a medicinal diploid (2n = 18) cross-pollinated perennial sub-shrub endemic to Greece (Christodoulakis et al. 2014; Papaefthimiou et al. 2014; Totta et al. 2017; Ganos et al. 2025). It is widely recognized for its hardiness and for its capacity to quickly establish itself in low-quality agricultural lands with degraded soils, including polluted mining sites as well as abandoned, overgrown, or fire-affected areas (Frazão et al. 2018). Another distinguishing trait of *C. creticus* is the seasonal leaf dimorphism, which the species has developed for adaptation to drought (Christodoulakis et al. 2014), along with the thick covering of the aerial parts (leaves, stems, and calyces) with glandular and non-glandular trichomes (Falara et al. 2010; Skorić et al. 2022). Between these, the single-stranded elongated capitate glandular trichomes produce and secrete a sticky resin, known as ‘ladano’ (Falara et al. 2010; Skorić et al. 2022), a rich source of pharmacologically important metabolites, mainly terpenoids, monoterpenes, sesquiterpenes, and labdane-related diterpenes (Demetzos et al. 1997; Falara et al. 2010; Papaefthimiou et al. 2014; Skorić et al. 2022; Papanikolaou et al. 2024; Ganos et al. 2025).

*C. creticus* ladano has been traditionally used as incense and aromatic agent, but also ladano exhibits a range of valuable pharmaceutical activities, like anti-inflammatory and antimicrobial activities against pathogenic bacteria and fungi (Papaefthimiou et al. 2014; Skorić et al. 2022; Ganos et al. 2025), along with the capacity to modulate immune cell functions (Papaefthimiou et al. 2014; Ganos et al. 2025). Furthermore, ladano possesses noteworthy cytotoxic and cytostatic actions towards several human leukemic and tumor cell lines (Chinou 2005; Hatziantoniou et al. 2006; Matsingou et al. 2006; Skorić et al. 2012; Papaefthimiou et al. 2014; Abu-Orabi et al. 2020; Lahcen et al. 2020; Ganos et al. 2025). These biological properties are primarily ascribed to labdane-related diterpenes (LRDs), with the most significant being manoyl oxide, 13-epi-manoyl oxide, 13(16),14-labdien-8-ol, labda-7,13-dien-15-ol, *ent-*3β-hydroxy-13-epi-manoyl oxide, *ent-*3β-acetoxy-13-epi-manoyl oxide, labda-7,13(E)-dien-15-yl acetate, labda-(13E)−8α,15-diol, and labda-(13E)−8α,15-yl acetate manoyl oxide (Anastasaki et al. 1999; Skorić et al. 2012, 2022; Papaefthimiou et al. 2014; Ganos et al. 2025).

The biosynthesis of LRDs takes place in the plastids of the capitate-type glandular trichomes of young leaves (Falara et al. 2010; Skorić et al. 2022; Papanikolaou et al. 2024) through the methylerythritol 4-phosphate (MEP) pathway and the action of class II terpene synthases (TPS) and decorating enzymes such as BAHD acyltransferases (Papanikolaou et al. 2024). Earlier studies in our group revealed the functional characterization of the trichome-specific class II diterpene cyclase genes, namely *copal-8-ol diphosphate synthase* (*CcCLS*) (Falara et al. 2010), two *labda-7,13(E)-dien-15-yl diphosphate* synthases (*endo-7,13-CPP, CcLDDS1 and CcLDDS2*), along with a gene encoding a BAHD acetyltransferase (*CcLAT*) capable of acetylating the aforementioned labdane-related diterpenes (Papanikolaou et al. 2024).

Characterization of plant germplasm diversity is usually performed by metabolomic profiling, although expedient, it is limited by the influence of the environmental conditions on the chemotypic expression (Sarrou et al. 2017). An alternative option to assess diversity is through genetic analysis with the use of molecular markers as inter simple sequence repeats (ISSR) (Paolini et al. 2009), which is a simple and cost-effective method for evaluating population structure and genetic relationships. The advantage of the ISSR markers is that they demonstrate high reproducibility, strong polymorphism, and stability, making them valuable for estimating evolutionary processes (Song et al. 2010; Peng et al. 2023). Although *C. creticus* specialized metabolism is well-documented, there is a lacuna of information concerning the genetic diversity of its populations, knowledge that is fundamental, as the evaluation and comprehension of genotypic relationships are prerequisites for a successful plant breeding program (Cobb et al. 2019). Though these approaches are beneficial for the development of novel cultivars with enhanced traits, targeted biosynthetic strategies, as modifying key structural genes or their regulatory transcription factors (TFs), are necessary (Alfieri et al. 2018; Venegas-Molina et al. 2021). To gain insights into the *C. creticus* diversity, this study aims to explore the genetic and chemical diversity of this species’ populations in Greece from north to south by administering genetic analysis with the employment of ISSR markers, by applying chemical profiling of LRDs, and by implementing transcriptomic analysis and thus to correlate the content of specific LRDs with related genes’ expression to identify key genes implicated in the biosynthesis of these compounds. Understanding the relationships among genetic diversity, chemical composition, and gene expression will enhance our knowledge of this medicinally important species and support its sustainable utilization and conservation.

## Materials and methods

### Plant material

Shoot cuttings (with leaves) of the last vegetative period were collected from 131 genotypes, originating from 7 populations from different geographic regions of Greece (authorization by the Greek Ministry of Environment and Energy, 175958/5915–22.11.2018) and transferred to the facilities of the Floriculture Laboratory (Department of Agriculture, Aristotle University of Thessaloniki Farm, Thermi, Greece). Then, the base of the cuttings was immersed (1 cm of the base) in 2,000 ppm rooting regulator K-IBA (potassium salt of indole-3-butyric acid; Sigma-Aldrich, St. Louis, MO, USA) solution, for 10 s and subsequently, transferred to plastic boxes containing perlite substrate (Isocon, Athens, Greece). The cuttings were maintained for four weeks in a fog-rooting unit at 95% RH and then were moved to 1 L pots containing a 3:1 peat (TS2; Klasmann, Geeste, Germany) to perlite (Isocon) substrate. After an eight-month growth period in the greenhouse, the plants were transferred outdoors to establish a *C. creticus* experimental field (Lat 40.53593 and Lon 22.99397). At the end, out of the 131 genotypes initially sampled for propagation, 91 were finally successfully rooted and established, and thus evaluated (Supplementary Table S1), each represented by 3 biological replications.

### DNA isolation, PCR amplification, and ISSR data analysis

DNA isolation from the 91 *C. creticus* genotypes was performed by NucleoSpin Plant II kit (Macherey Nagel, Düren, Germany) according to the manufacturer’s protocol. The DNA quantity and quality were measured by a NanoDrop™ 2000c (Thermo Fisher Scientific Inc., Waltham, MA, USA), and by agarose electrophoresis, respectively, and the DNA samples were diluted to a 20-ng/μL working solution.

PCR amplification for ISSR analysis was performed by a ProFlex thermocycler (Life Technologies, Thermo Fisher Scientific) in a reaction volume of 10μL using a KAPA2G fast PCR kit (Kapa Biosystems, Cape Town, South Africa) with the following condition: initial denaturation step at 95 °C for 3 min was followed by 30 cycles of 15 s denaturation at 95 °C for 15 s, annealing at Tm°C of each primer pair for 15 s and extension at 72 °C for 45 s. This was followed by a final extension step at 72 °C for 3 min (Kostas et al. 2021; Ntoanidou et al. 2024). Eleven ISSR markers that were reproducible and polymorphic were used for the estimation of genetic diversity (Supplementary Table S2). For the analysis, only bands unambiguously scored across all the tested genotypes for each marker were used to create a binary matrix where the presence or absence of a band was denoted by 1 or 0, respectively (Fig. S1). For every primer locus, polymorphism information content (*PIC*) was determined from the band patterns using *PIC* = 1 − Σ(Pi)^2^, where Pi corresponds to the occurrence frequency of each band across the genotypes evaluated (De Riek et al. 2001; Kostas et al. 2021). Data analysis was performed by GenAlEx v.6.501 (Peakall and Smouse 2006; Smouse and Peakall 2012) and Mega X software (Molecular Evolutionary Genetic Analysis) (Kumar et al. 2018).

### Labdane-related diterpenes determination and quantification

Leaves of the first three developmental stages (S1-S3, 0.1–2.5 cm) were harvested, instantly frozen in liquid N_2,_ and kept at − 80 °C until further analysis (Falara et al. 2010). For each of the 91 genotypes, three biological replicates (three individual plants per genotype) were tested. For the extraction of the LRDs an adapted protocol was created by similar studies on *Crocus sativus*, *Cistus monspeliensis,* and various Sardinian *Cistus* species (Rivoal et al. 2010; Aliakbarzadeh et al. 2016; Mastino et al. 2017), where 1 g of leaves from each biological replicate was ground with a mortar and pestle in the presence of liquid N_2_ and extracted with 10 mL of high purity anhydrous *n*-hexane (GC–MS grade, Sigma–Aldrich). The extracts were transferred to glass vials, vortexed for 1 min, and placed in a Turbovap LV nitrogen deaerator (Caliper Life Sciences, Hopkinton, MA, USA) until the solvent was completely removed. The dry residue was redissolved by adding 5 mL of *n*-hexane. As an internal control, 200 μL of nonadecane (C19) hydrocarbon solution (Fluca, Buchs, Switzerland) (ca. 200 μg/mL) was added to each sample. The extracts were then vortexed for 1 min and centrifuged for 5 min at 10,000 g. The resulting supernatant was filtered through a 0.20-µm filter twice and subsequently concentrated to approximately 0.5 mL using a Turbovap LV. The final volume was first adjusted to 1 mL and then placed into a glass vial and stored at − 20 °C until further use.

Labdane-related diterpenes were detected by using a GC–MS-QP2010 Ultra type gas chromatography system (Shimadzu Corp., Kyoto, Japan) containing two detectors, a flame ionizer (FID, Flame Ionization Detector) and a mass spectrometer (MS, Mass Spectrometry). A DB-5MS capillary column of dimensions (30 m × 0.25 mm, 0.25 μm film thickness) was used in splitless mode for the analysis. The temperature of the introducer and probe was at 230 °C and 200 °C, respectively. The carrier gas was helium with a flow of 1.04 mL/min. The mass spectrum was obtained in scan mode, while the qualitative analysis was done using the libraries FFNSC GC/MS Ver. 1.3, Metabolite Component Database by Shimadzu, Wiley 7, NIST 11, and NIST 11s, combined with the Kováts index (Supplementary Table S3) (Van Den Dool and Kratz 1963; Adams 2017). A formulation containing hydrocarbons from C7 to C30 (Fluca) was used to check the quality of the chromatograms. Each chromatogram ran a 23-min program, which followed the following gradient: the oven temperature was initially 50 °C and remained at this temperature for 3 min, then gradually increased at a rate of 14 °C/min up to 300 °C and remained at this temperature for 3 min. As the primary objective of this study was the comparative evaluation of the genotypes rather than absolute quantification, the levels of the detected compounds were estimated by using C19 as an internal control, following methodologies previously described in the literature (Rivoal et al. 2010; Aliakbarzadeh et al. 2016; Mastino et al. 2017).

### Multiple regression analysis between ISSR markers and LRDs content

Among the ISSR marker bands and the amount of the main substances identified in the *C. creticus* leaves of each genotype, the correlations were calculated by multiple regression analysis (MRA) using a stepwise linear regression. The multiple correlation coefficient *R*^2^, squared by *r*, gives the % probability that one event will occur relative to another, and the beta value is defined as the standard regression coefficient (Virk et al. 1996; Kar et al. 2008; Ganopoulos et al. 2011).

### Genotype selection for transcriptomic analysis

Two genotypes of *C. creticus* from the population of Chalkidiki, namely C1 and C18 exhibiting the lowest and the highest LRDs contents, respectively, were separately selected for a more thorough chemical analysis in the three developmental stages (S1, S2, S3). For this a different extraction and GC–MS analysis was followed. In detail, 500 mg from frozen leaves of each developmental stage, was pulverized in liquid nitrogen and mixed with 5 mL of *n*-hexane (GC–MS grade, Sigma–Aldrich), vortexed vigorously, and the extraction proceeded under sonication for 15 min at room temperature. The extracts were centrifuged for 10 min at 1800 g (4 °C). The extraction was repeated three times, and after combining the hexane phase, anhydrous sodium sulfate was added to absorb water, and the extracts were filtered using Whatman paper. The *n*-hexane evaporated with gas nitrogen until crude complete dryness of each sample. The crude extracts were reconstituted in 2 mL *n*-hexane containing nonadecane as an internal standard. The extracts were injected directly, and the data were expressed as the means of three biological replicates per leaf sample.

GC–MS analysis of the *n*-hexane leaf extracts was performed using a Shimadzu 17 A Ver. A chromatograph, equipped with an AOC20i autosampler (Shimadzu Corp), and interfaced with QP-5050A mass spectrometer, which was supported by GC/MS Solution ver. 1.21 software. The separation of the compounds was carried out on a capillary HP-5MS (30 m, 0.25 mm, 0.25 m column) (Agilent Technologies Inc.) under the following thermal program: the oven temperature was raised from 55 °C (hold time 3 min) up to 160 °C with a rate of 12 °C min^–1^, followed by an increase of 3 °C min^–1^ up to 230 °C, and finally by 20 °C min^–1^ until 325 °C remaining for 15 min (total run time 56 min). The injection temperature was set at 230 °C in splitless mode, and for the analysis, 2 μL of the samples was injected. The interface line was set at 300 °C, ion source at 200 °C, EI mode: 70 eV, scan range: 41–450 amu, and scan time at 0.50 s. Helium was used as the carrier gas with a constant flow rate of 1 mL min^–1^. The identification of the produced peaks was conducted through comparative analysis of their spectra with those from commercial MS libraries (NIST, Wiley), the Flavour and Fragrance Natural and Synthetic Compounds GC–MS library (FFNSC 2, 2012) and through comparison with literature (Adams 2017; Papanikolaou et al. 2024) (Fig. S2; Supplementary Table S4). The relative content of each compound was calculated using nonadecane as an internal standard, and the results were expressed as means of three biological replicates.

### Transcriptomic analysis

Total RNA was extracted from the S1 developmental stage of young leaf tissue (0.1–1 cm) of three biological replicates (three individual plants per genotype) of two selected genotypes (C1, C18) using the Spectrum Plant Total RNA kit (Sigma-Aldrich) as per the manufacturer’s protocol. The integrity and quality of the extracted RNA were initially assessed by NanoDrop™ 2000c (Thermo Fisher Scientific), agarose gel electrophoresis, and subsequently were sent to Beijing Genomics Institute (BGI, Shenzhen, China) where the RNA-sequencing was performed by a DNBseq platform with a paired-end sequencing length of 100 bp. A total of six libraries were constructed, and raw data were submitted to the National Center for Biotechnology Information (NCBI) Sequence Read Archive (SRA) under BioProject accession number PRJNA1378087. The raw reads were filtered to remove adaptor sequences, reads with unknown bases (N ≥ 5%), and low-quality reads. After filtering, the reads were called clean reads and stored in a FASTQ format. For plant species without a reference genome, clean reads were assembled after sequencing to get a reference sequence for subsequent analysis. After reads’ filtering, Trinity v2.0.6 software was applied to perform *de-novo* assembly with clean reads, and Tgiclv2.0.6 software was used to cluster transcripts, remove abundance, and to get Unigenes. Annotation of transcript to the seven functional databases (Nr, Nt, GO, KOG, KEGG, SwissProt, and InterPro) was performed with Blast v2.2.23 software and parameters set at default. Results of the GO annotation were generated using Blast2GO v2.5.0 and Nr, InterPro annotation with InterProScan5 v5.11–51.0 with parameters set at default.

### Differential expression of gene analysis (DEG)

To quantify gene expression, clean reads were mapped to the assembled Unigenes using the Bowtie2 v2.2.5 software, while gene expression levels were calculated using the RSEM v1.2.12 software with default parameters. The resulting expression values were reported as Fragments Per Kilobase of transcript per Million mapped reads (FPKM). Differentially expressed genes (DEGs) were identified using the DESeq2 package in R Bioconductor, applying default parameters for the analysis. Genes with *p*-adjusted ≤ *0.05* and log2 fold change ≥ 1 were considered up-regulated, whereas those with log2 fold change ≤ −1 were considered down-regulated.

To identify putative transcription factors (TFs) that may participate in the regulation of the LRDs biosynthesis, an in-depth analysis was conducted in the Nr functional database, and significantly up-regulated genes of *C. creticus* transcriptome were revealed. A regulatory network in Cytoscape (v 3.10) (Shannon et al. 2003) was constructed between TFs and specific labdane compounds (sclareol, labda-(13E)−8α,15-diol, manoyl oxide, 13-epi-manoyl oxide, *ent*−3β-hydroxy-13-epi-manoyl oxide, *ent-*3β-acetoxy-13-epi-manoyl oxide), and between TFs and labdane biosynthetic gene pairs, exhibiting a significant Pearson correlation coefficient (r ≥ 0.95, p< 0.05). Furthermore, a Spearman correlation was performed between the expression profiles of the labdane biosynthetic genes and these specific labdane compounds.

### Quantitative real-time PCR

cDNA was synthesized using the Superscript® III Reverse Transcriptase kit (Invitrogen, Thermo Fisher Scientific) as per the manufacturer’s protocol in 1 μg RNA for each sample. For validation of the RNA-seq results, qRT-PCR was performed for genes involved in the biosynthetic pathway of LRDs on an ABI 7500 Fast Real-time PCR system (Applied Biosystems) using a Kapa™ SYBR FAST qPCR Kit (Kapa Biosystems) and gene-specific primers (Supplementary Table S5) according to Papanikolaou et al. (2024) at a reaction volume of 10 μL. The cycling conditions for qRT-PCR were the following: 20 s/95 °C for one cycle, (3 s/95 °C, 30 s/66 °C, 20 s/72 °C) for 40 cycles, followed by a single melting curve cycle: 15 s/95 °C, 1 min/60 °C, temperature increase 60–95 °C at a rate 1 °C s^–1^, 15 s/95 °C. The relative transcript abundance was calculated using the 2^−ΔCt^ method where ΔCt represents the Ct _Target gene_ − Ct _Reference genes_ [Ct _Reference genes_ = (Ct _actine_ + Ct _elongation factor_)/2].

### Variant calling and annotation

Coding regions were identified using TransDecoder v5.5 (Haas et al. 2013), which predicts likely protein-coding sequences based on ORF length, coding potential, and homology evidence. First, long open reading frames were extracted with TransDecoder. LongOrfs, and candidate coding regions were subsequently refined using TransDecoder. Predict to determine the most probable CDS for each transcript in the *de-novo* assembly. The resulting GFF3 was used as structural gene models for variant annotation. Trimmed reads passing the filtering criteria (average quality ≥ Q20, minimum length ≥ 18 bp) were mapped to the *de-novo* transcriptome assembly using Hisat2 with default parameters. PCR duplicates were marked using Picard (v 2.22.8), and multi-mapped reads were removed using samtools (v1.11) (Li et al. 2009). Because RNA aligners have different conventions than DNA aligners, we reformatted some of the alignments that span introns for HaplotypeCaller. SplitNCigarReads tool splits reads with N in the cigar into multiple supplementary alignments and hard clips mismatching overhangs, reassigning mapping qualities for good alignments to match DNA conventions. Genome Analysis ToolKit (GATK) was used for variants calling and classification into single nucleotide polymorphisms (SNPs) or InDels only, performed using HaplotypeCaller, CombineGVCFs, GenotypeGVCFs, Select Variants, and Variant Filtration. Variants belonging to individual samples were extracted using bcftools (v1.11), while variant annotation was conducted using SnpEff (v 4.3) (Cingolani et al. 2012).

### Statistical analysis

Normality of the values was checked by the Levene test (*a > 0.05*) and means comparison for the data obtained from the GC–MS-QP2010-MS analysis was conducted by Duncan test at *a* = *0.05*. The aforesaid statistical analyses, as well as Hierarchical Cluster Analysis, PCA, and MRA were performed with SPSS 28 (IBM, Armonk, NY, USA).

## Results

### Genetic diversity analysis

Genetic analysis of the 91 *C. creticus* genotypes revealed an average of 43.361% polymorphism at the population level of 27.43% in Agrinio (Ag) and 60.18% in Floria (F) population (Table 1). The values of Shannon’s index (I) and Nei’s gene diversity (h) showed similar trends with an average at the population level of 0.217 and 0.143, respectively. Specifically, I value ranged from 0.166 in Agrinio (Ag) to 0.304 in Floria (F) populations, and h values ranged from 0.114 in Agrinio (Ag) to 0.199 in Floria (F) populations, indicating that the gene diversity in the population of Floria (F) was the richest among the 7 populations. Tested markers UBC812, UBC810, and UBC808 had the highest percentage of polymorphism (%) and PIC values. In contrast, UBC821 had the lowest values in the corresponding areas (Supplementary Table S2).

**Table 1:** Genetic diversity of *C. creticus* populations and species based on the results of ISSR markers

Population	Na	Ne	I	h	*p* (%)
Chalkidiki (C)	1.319	1.205	0.199	0.127	48.67
Floria (F)	1.469	1.33	0.304	0.199	60.18
Sises (S)	1.407	1.272	0.252	0.164	55.75
Akrotiri (A)	1.336	1.249	0.234	0.152	47.79
Manoliopoulo (M)	1.071	1.194	0.173	0.116	30.97
Chios (Ch)	1.035	1.226	0.191	0.13	32.74
Agrinio (Ag)	0.991	1.2	0.166	0.114	27.43
Mean	1.233	1.239	0.217	0.143	43.361

*Na* number of alleles, *Ne* effective number of alleles, *h* Nei’s gene diversity, *I* Shannon’s information index, *P* percentage polymorphism bands

Analysis of molecular variance exposed highly significant genetic intra-population differences with a percentage of 71% ( p< 0.001). Furthermore, AMOVA disclosed highly significant differences among the 7 populations, with 29% of the total gene diversity attributed to the differences among populations (Φ_ST_ = 0.294), supporting the results of Nei’s gene diversity and Shannon’s index (Table 2).

**Table 2:** Analysis of molecular variance (AMOVA) showing the partitioning of genetic variation within and among the 7 *C. creticus* populations based on ISSRs data

**Source**	**df**	**SS**	**MS**	**Est. Var**	**%**
Among Pops	6	315.691	52.615	3.724	29%
Within Pops	84	760.309	9.051	9.051	71%
Total	90	1076.000		12.775	100%
Stat	Value	*p* (rand ≥ data)			
Φ_ST_	0.291	0.001			

*df* degree of freedom, *SS* sum of squares, *MS* mean squares, *Est. Var.* estimate of variance, *%* percentage of total variation, *U PT* PhiPT. *Φ*_*ST*_ fixation index

**Fig. 1:**
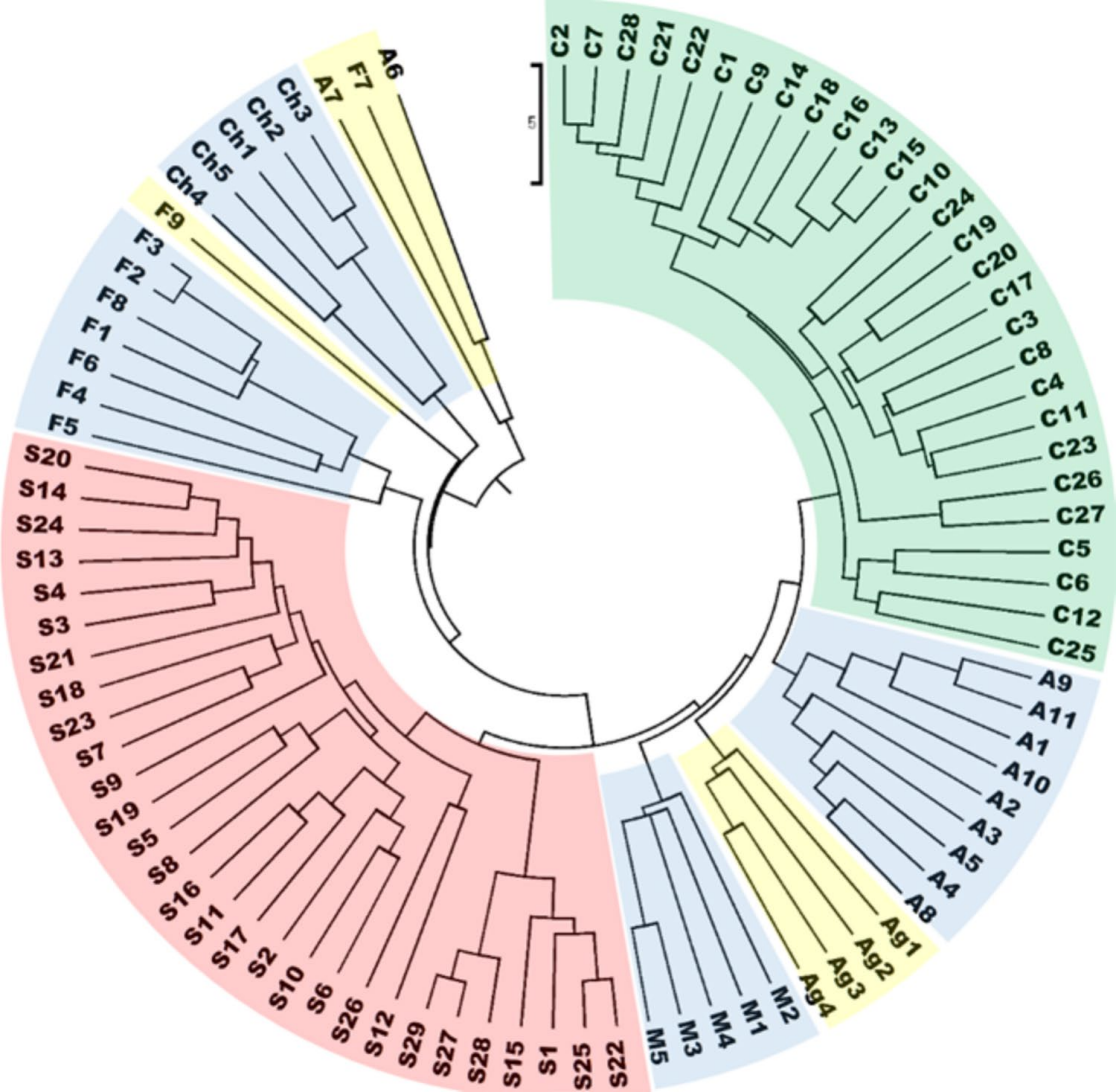
A UPGMA dendrogram illustrating the genetic relationships among 91 *C. creticus* genotypes from seven different populations based on Nei’s genetic distance calculated from 11 ISSR molecular markers. The abbreviations of the 9 *C. creticus* populations are: Chalkidiki (C), Sises (S), Akrotiri (A), Floria (F), Chios (Ch), Agrinio (Ag) and Manoliopoulo (M)

To visualize the genetic structure of *C. creticus* populations, multiple genetic clustering analyses were performed. A UPGMA dendrogram was used to disclose the relatedness among the 7 populations and 91 genotypes and grouped the *C. creticus* genotypes into two highly distinct and homogenous clades, one of Chalkidiki (C) and one of Sises (S), and many sub-clades from the other regions. In the clade with pink color, there are all the genotypes from Sises (S1–S29), and in the clade with green color, there are all the genotypes from Chalkidiki (C1–C28) without the appearance of other genotypes. The remaining populations of the other five regions were grouped into a more complex series of several mixed smaller sub-clades, a sub-clade with two genotypes from Akrotiri (A6 and A7) and one from Floria (F7) and two bigger sub-clades with the genotypes from Chios and from Floria. Another clade is comprised of three sub-clades, one from Manoliopoulo (M), one from Agrinio (Ag) and one from Akrotiri (A), suggesting closer genetic relationships among them. It is obvious that the genotypes from the same region are grouped within the same clades, which indicates that the genotypes stand out in most cases according to their geographical distribution (Fig. 1).

Principal Coordinates Analysis (PCoA) provided a complementary visualization of the genetic structure of *C. creticus* genotypes, with the first two coordinates representing 19.59% of the total variation (Fig. S3). The PCoA plot organized the genotypes into three separate groups, corroborating and complementing the UPGMA results, with Chalkidiki and Sises populations to form two separate clusters. The third cluster is a mix of the remaining five populations, with the Akrotiri population scattered across the plot, indicating a high degree of heterogeneity. For the populations from Chios, Agrinio, and Manoliopoulo a small number of individuals were used, because the cuttings from some genotypes of these areas were not rooted, so no safe conclusions can be drawn about their genetic composition.

**Fig. 2:**
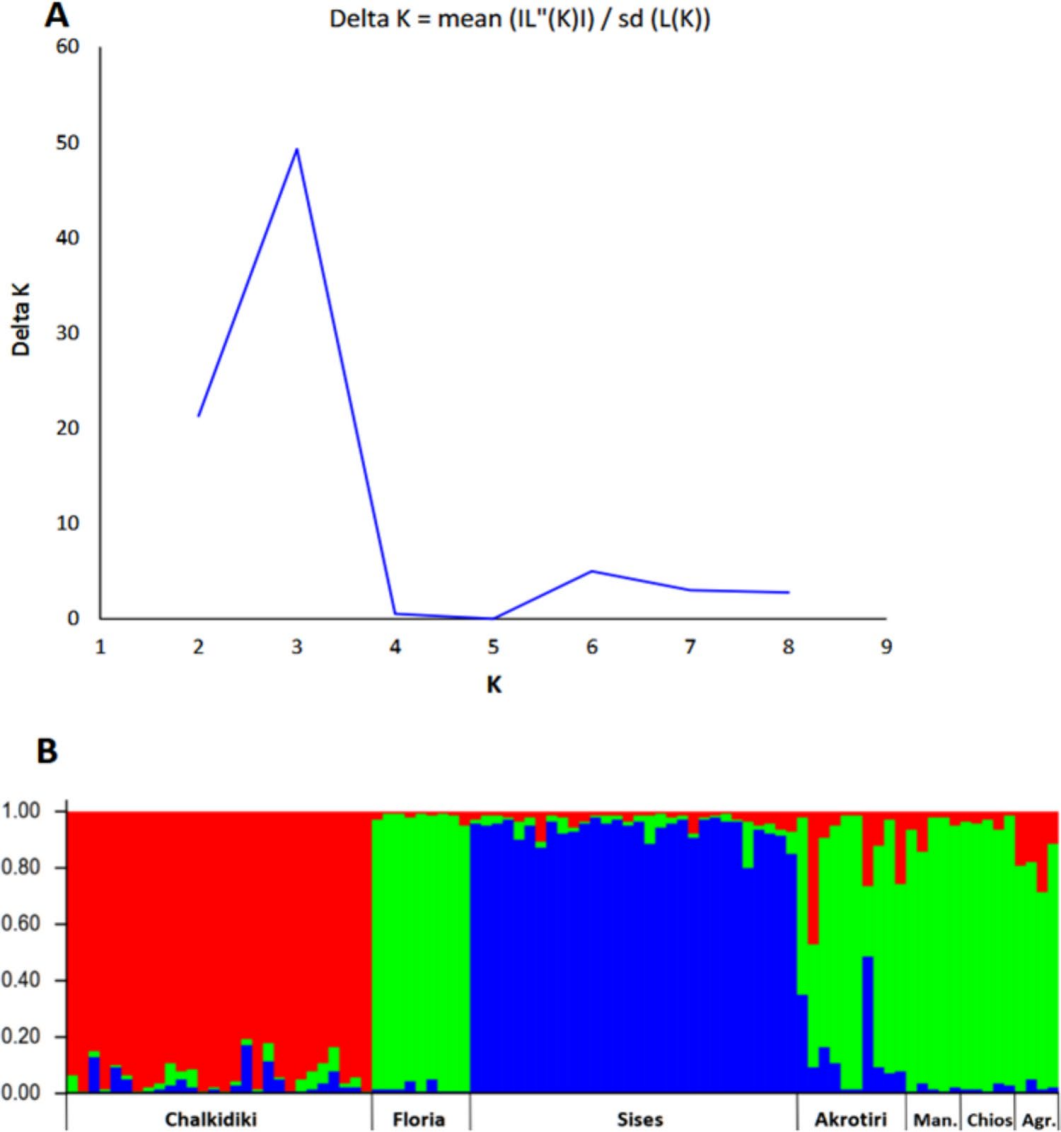
STRUCTURE Analysis of 91 genotypes and 7 populations of *C. creticus* based on 11 ISSRs markers.** A** Calculation of *ΔK* with highest range equal to 3 suggesting the categorization of genotypes in three clusters, red, green, and blue. **B** Genetic relationship of 91 *C. creticus* subsp. *creticus* genotypes. Each vertical line represents one of the 91 genotypes and its probability of belonging to three groups (*ΔK* = 3 clusters) and 7 populations. In red are the genotypes from Chalkidiki, in blue the genotypes from Sises, and in green the other genotypes. Genotypes with red, green, and blue color together indicate possible transfer of genetic material among regions

STRUCTURE analysis based on a Bayesian model with the highest likelihood of the data obtained for *ΔK* = 3 (Fig. 2A) (Evanno et al. 2005), provided the fullest picture for the *C. creticus* populations’ structure. The analysis illustrated low levels of admixture for the Floria, Sises, Manopoulio, Chios, Chalkidiki, and Agrinio populations, whereas moderate levels were observed in the Akrotiri population (Fig. 2B; Fig. S4). The results of STRUCTURE analysis coincide perfectly with the results of the PCoA, where the genotypes are also genetically divided into three groups.

### Labdane-related diterpenes (LRDs)

Sclareol oxide was the first LRD detected, with genotype Ch1 (Chios) showing the highest concentration (68.29 µg/g leaf). At the population level, Chalkidiki had the highest mean (25.17 µg/g leaf), followed by Sises (20.25 µg/g leaf). Manoyl oxide was most abundant in Chalkidiki (31.89 µg/g leaf) and Sises (29.77 µg/g leaf), with genotypes C13, C14, and C16 from Chalkidiki showing the highest values. For 13-epi-manoyl oxide, C6 (Chalkidiki) had the highest yield (323.89 µg/g leaf), followed by S7 and S19 from Sises; these two populations also had the highest averages (Fig. S5; Supplementary Table S6).

The compound 13(16),14-labdien-8-ol was highest in Chalkidiki genotypes C14, C13, C23, and C10, while Sises and Chalkidiki populations showed significantly higher means. Abienol production was greatest in C13 and C16 (Chalkidiki) and S5 and S20 (Sises), with both populations again showing the highest values. Sclareol was most abundant in S28 (Sises), and Sises and Chalkidiki populations had the highest means. Labda-7,13-dien-15-ol peaked in F6 (Floria) and C23 (Chalkidiki), with Sises and Akrotiri populations showing the highest levels (Fig. S5; Supplementary Table S6).

**Fig. 3:**
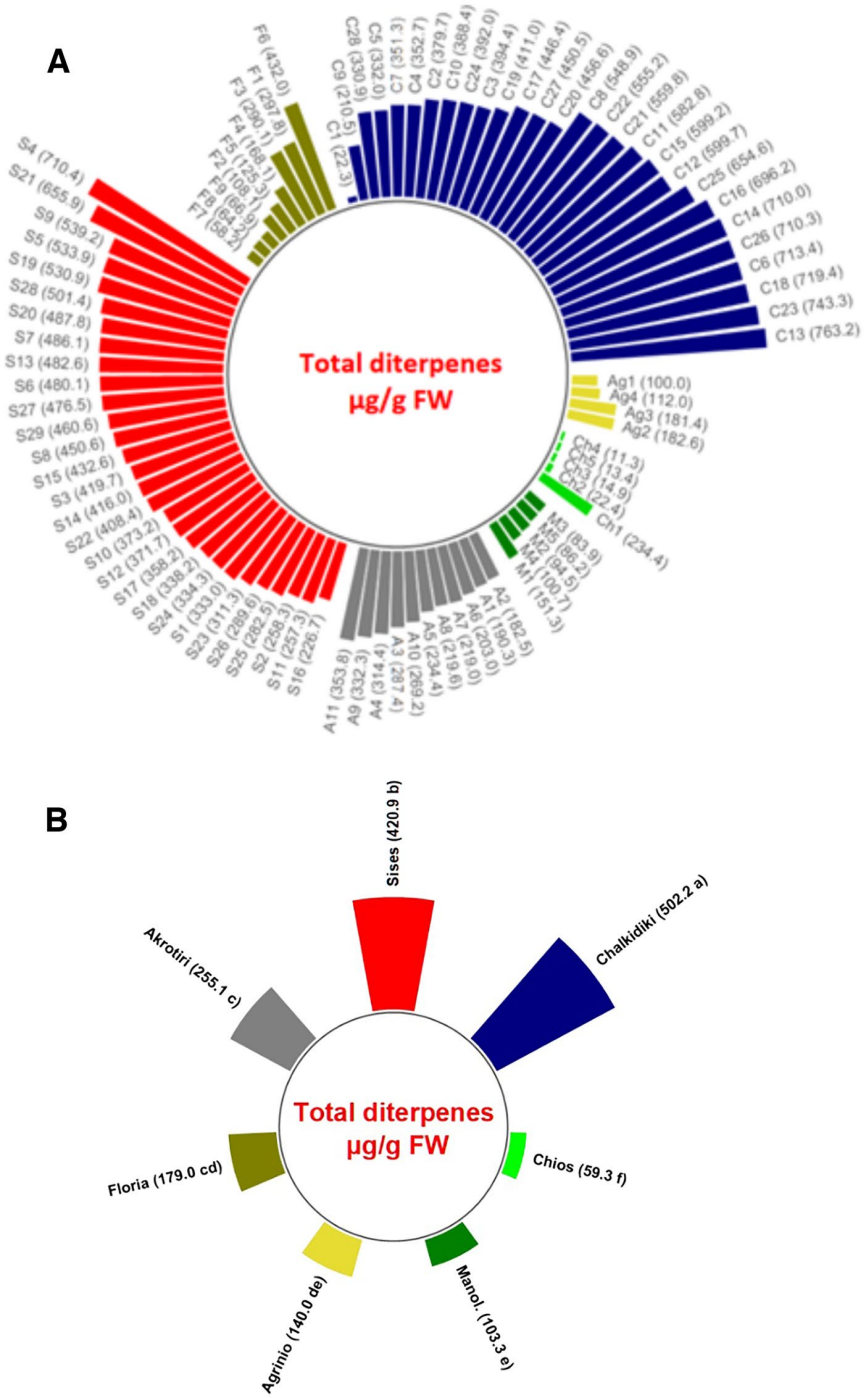
Concentration of total labdane-related diterpenes μg/g fresh young leaves of *C. creticus* growing in outdoor plant collection. **A** allocation of the 91 genotypes (*n* = 3, from sampling in three different plants–clones of the same genotype) of *C. creticus*. **B** the 9 populations Chalkidiki, Sises, Akrotiri, Floria, Chios, Agrinio and Manoliopoulo of *C. creticus* in descending order

For *ent*−3β-hydroxy-13-epi-manoyl oxide, S21 and C14 showed the highest concentrations, and Chalkidiki had the greatest population mean. *Ent*−3β-acetoxy-13-epi-manoyl oxide was highest in several Chalkidiki genotypes (C18, C26, C14, C13, C16, C25), with Chalkidiki again leading at the population level. Labda-7,13-dien-15-yl acetate was highest in F6 (Floria) and C23 (Chalkidiki), while Akrotiri, Sises, and Manoliopoulo populations showed the greatest averages. Labda-(13E)−8α,15-diol was highest in C12, C13, and S28, and Chalkidiki, Sises, and Akrotiri populations had the highest means. Labda-(13E)−8α,15-yl acetate peaked in C23 (Chalkidiki), with Chalkidiki and Sises populations showing the highest values (Fig. S5; Supplementary Table S6). Overall, genotype C13 produced the highest total LRDs (763.20 µg/g leaf), while Chalkidiki was the richest population (502.20 µg/g leaf) (Fig. 3).

**Fig. 4:**
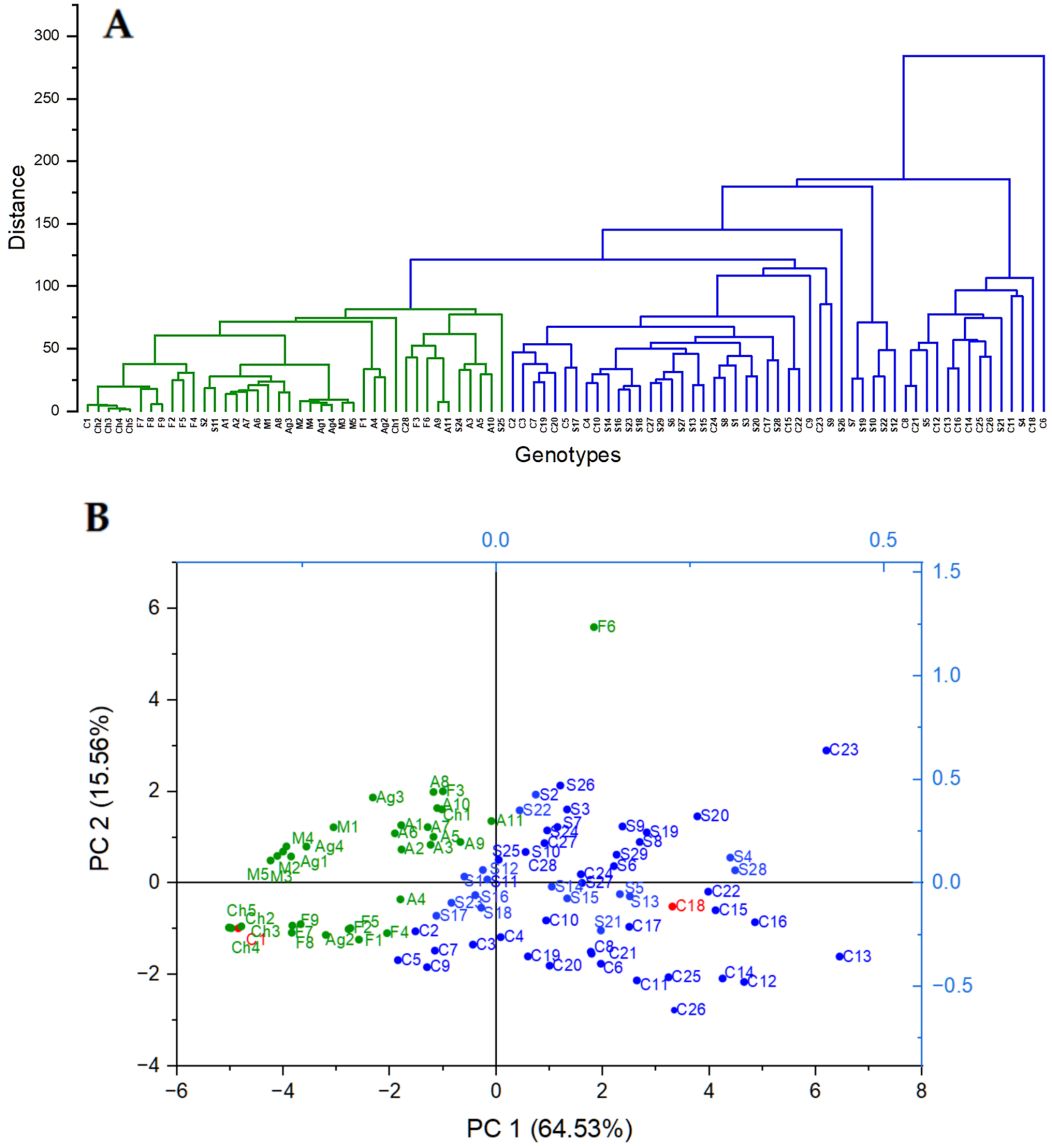
**A** Hierarchical Cluster Analysis (HCA) based on the chemical analysis of 12 labdane-type diterpenes. **Β** Principal Component Analysis (PCA) based on the data of 12 labdane-related diterpenes produced from *C. creticus* young fresh leaves. In both **A** and **B** types of analysis, the genotypes are divided into two groups: the first group with blue color includes the most *C. creticus* genotypes from Sises and Chalikidiki and the second group with green color includes most genotypes from the other locations. The two genotypes of *C. creticus*, C1 and C18, selected for transcriptomic analysis as LRD-poor and LRD-rich genotypes, respectively, are colored in red in PCA (**B**)

To investigate the chemical diversity of the examined populations, a Hierarchical Cluster Analysis (HCA) and Principal Component Analysis (PCA) were performed based on the data of the chemical profile of each genotype. The HCA dendrogram disclosed the partitioning of the genotypes into two main clusters or chemotypes, the first one consists of the populations of Chalkidiki and Sises, whereas the second cluster comprises the remaining five populations (Fig. 4A). To further elucidate the structure of the chemotypes, PCA was conducted, where the first two components explain 80.09% of the total variation and separate the genotypes into three distinct clusters. The PC1 captures the majority of the variance and perfectly separates the genotypes into two groups, the green, which is a mixture of the Chios, Agrinio, Manoliopoulo, Floria and Akrotiri; the blue group contained the Sises population and Chalkidiki. The mixed-origin genotypes (green group) that were exclusively located in the negative region of PC1 suggest that these populations were the least productive for LRD, while the Sises and Chalkidiki genotypes, which occupied the positive region were the most productive (Fig. 4B).

An alternative way to visualize the abundance of each LRD compound is illustrated by a heatmap in which red color indicates a high abundance, orange a medium and green a low one (Fig. S6). The heatmap revealed distinct chemical profiles for each population; genotypes from Chalkidiki exhibited the highest productivity across most compounds, followed by those from Sises and Akrotiri. Conversely, the lowest production levels were observed in genotypes from Manoliopoulo, Chios, Agrinio, and Floria (Fig. S6).

### Multiple regression analysis

To identify possible relationships between ISSR markers and LRDs, a multiple regression analysis was performed, where the production of the total diterpenes (Fig. S7A, B) was positively correlated with the UBC812_980_, UBC808_800_, UBC811_400_, UBC811_970,_ and UBC808_1450_ and negatively with the UBC818_1400_, UBC810_500_ and UBC811_450_ markers with a probability of 63.9% (R^2^) (Table 3; Fig. S8). Among the positive predictors, the UBC808_800_ exerted the strongest correlation based on the *β* (0.332) and *t* (4.671) values, while UBC8081_450_, a significant positive predictor, has lower values of *β* (0.173) and *t* (2.491). The significance of all eight markers was confirmed by the *p*-values ranging from < 0.001 to 0.027.

**Table 3:** Correlations between bands of ISSRs (UBC) molecular markers and the concentration of total diterpenes of *C. creticus* genotypes, as revealed by stepwise multiple regression analysis (MRA) and coefficients

ISSR markers (alleles)	*r*	*R*^*2*^	Standard error	Standardized beta (*β*) coefficients	*t *value	*p *value
Total diterpenes						
UBC812_980_	0.497	0.247	0.238	0.202	2.531	0.013
+ UBC808_800_	0.608	0.37	0.356	0.332	4.671	0
+ UBC811_400_	0.675	0.456	0.437	0.173	2.257	0.027
+ UBC818_1400_	0.717	0.514	0.492	− 0.292	− 4.067	0
+ UBC810_500_	0.75	0.562	0.536	− 0.297	− 3.923	0
+ UBC811_450_	0.767	0.589	0.559	− 0.185	− 2.472	0.016
+ UBC811_970_	0.782	0.611	0.579	0.183	2.618	0.011
+ UBC808_1450_	0.799	0.639	0.603	0.173	2.491	0.015

It should be noted that ISSR markers are dominant and multilocus, and therefore the observed associations with LRD content should be interpreted with caution. Given the diversity of the populations included in this study, the regression relationships may partly reflect population structure rather than direct marker–trait associations. Consequently, the identified markers should be considered preliminary, and further validation using codominant marker systems and larger populations would be required to confirm their transferability.

### Selection of *C. creticus* genotypes for RNA-sequencing

**Fig. 5:**
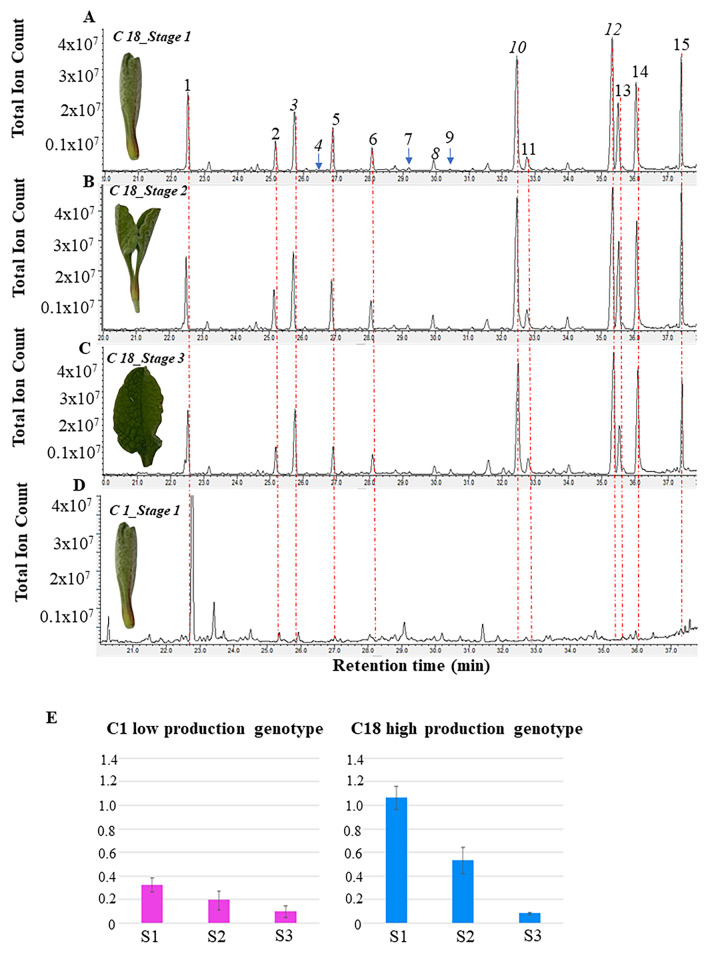
Selection *C. creticus* genotypes for RNA-sequencing based on metabolic profiling and gene expression. **A–C** GC–MS chromatograms of leaf extracts from genotype C18 at developmental stages S1 (**A**), S2 (**B**), and S3 (**C**). **D** GC–MS chromatogram of C1 genotype at the S1 developmental stage. **E** Relative expression level of the *CLS* gene in the C1 and C18 genotypes across leaf developmental stages (S1, S2, S3). Error bars represent ± SE (*n* = 3). The numbers in the chromatograph peaks denote the compounds: (1) nonadecane; (2) manoyl oxide; (3) *ent*−13-epi-manoyl oxide; (4) *ent*-kaur-16-ene; (5) selina-4,11-diene; (6) 13(16),14-labdien-8-ol; (7) neoabienol; (8) cis-abienol; (9) sclareol; (10) *ent*−3β-hydroxy-13-epi-manoyl oxide; (11) labda-7,13 (E)-dien-15-ol; (12) *ent*−3β-acetoxy-13-epi-manoyl oxide; (13) labda-7,13 (E)-dien-15-yl acetate; (14) labda-13(E)-ene-8a,15-ol; (15) labda-13(E)-ene-8a,15-ol acetate. Genotype C1 was analyzed in all three developmental stages, but only the S1 chromatogram is shown as S2 and S3 profiles were comparable

It was previously shown that *ent-*3β-acetoxy-13-epi-manoyl oxide was the most potent constituent in *C. creticus* resin against pathogenic bacteria (Skorić et al. 2022). For this, two *C. creticus* genotypes*,* C1 and C18, were selected for transcriptomic analysis based on their contrasting *ent*−3β-acetoxy-13-epi-manoyl oxide content (Figs. S5 9 A; Supplementary Table S6). While initial metabolite screening for selecting genotypes high in labdane diterpenes content, a pool of three developmental stages, namely S1 + S2 + S3 (Fig. 3A), was utilized, in contrast, for the RNA-seq analysis, we repeated the chemical analysis by focusing on diverging genotypes and single stages rich and poor in *ent-3β-acetoxy-13-epi-manoyl oxide* content. GC–MS profiling revealed that *ent*−3β-acetoxy-13-epi-manoyl oxide reached its maximum abundance in the S1 developmental stage of the C18 genotype, fact that was further confirmed by the significantly higher transcript levels of *CLS* gene in the same developmental stage (Fig. 5A–E). It should be noted that C1 genotype was also chemically analyzed across all three developmental stages; however, only stage S1 is presented in Fig. 5, since S2 and S3 stages were similar to S1.

### Overall transcriptome analysis of *C. creticus*

To investigate the differential transcriptomic profiles, as shown above, RNA-sequencing was conducted on the C1 and C18, low and high -*ent*−3β-acetoxy-13-epi-manoyl oxide- genotypes, respectively. After quality filtering, the C1 and C18 (deep-sequenced) genotypes generated 9.3–12.58 Gb and 28.01–32.34 Gb of high-quality data, respectively (Supplementary Table S7). A *de-novo* assembly was performed by filtering the reads, and Trinity software was used for the *de-novo* assembly of the clean reads, in order to obtain the reference sequence for further analysis. The average length of transcripts for all samples ranged between 633 and 786 bases, while the N50 value ranged from 1,008 to 1,426 bases (Supplementary Table S8). Tgicl software was used on clustered transcripts to remove abundance and retain Unigenes. In the present work, since there were more samples, Tgicl was utilized to the Unigenes of each sample, providing us with a single list named “All-Unigenes”. Consequently, after assembly of the sequences and filtering of all samples, 173,778 Unigenes were obtained, with total average length 1,031 bp and N50 1,872 bp (Supplementary Table S9).

**Fig. 6:**
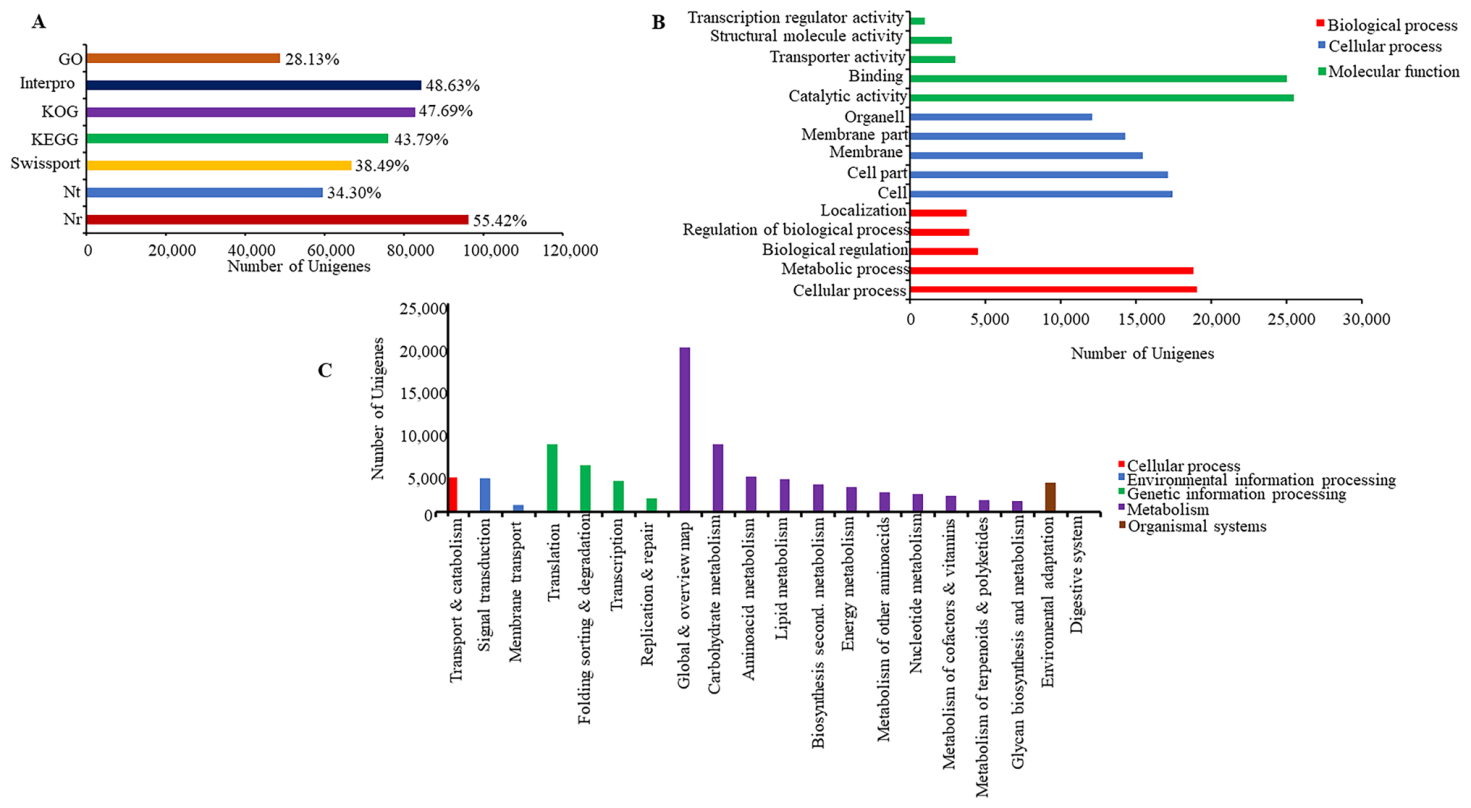
Gene annotation of *C. creticus* transcriptome. **A** Statistical results of mapping in the seven different databases. **B** Main categories of Biological Processes, Cellular Component and Molecular Function of the GO database. **C** Distribution of the number of Unigenes in the five functional categories of the KEGG database

Seven functional databases, namely Nr, Nt, SwissProt, KEGG, KOG, InterPro and GO were harnessed to functional mapping of 173,778 Unigenes. In total, 110,658 Unigenes (63.68%) have been mapped to at least one database, while a number of 20,218 Unigenes (11.63%) have been mapped to all 7 aforementioned databases, with Nr having the highest number of hits (96.316, 55.42%) (Fig. 6A). Among these transcripts, the 48,879 were linked to Gene Ontology (GO) terms including molecular function (MF), cellular component (CC), and biological process (BP), with the BP as the majority of GO terms (Fig. 6B). Regarding KEGG, the 76,095 Unigenes were further assigned into the five basic KEGG categories, in which metabolism occupies most of the *C. creticus* cell functional categories, while secondary metabolism in which the genes of interest of the present study occupy a substantial portion. It is of interest that the metabolism of terpenoids and polyketides pathway protrudes in this category with 1,383 transcripts (Fig. 6C).

### Expression profile and differential gene expression analysis (DEG)

**Fig. 7:**
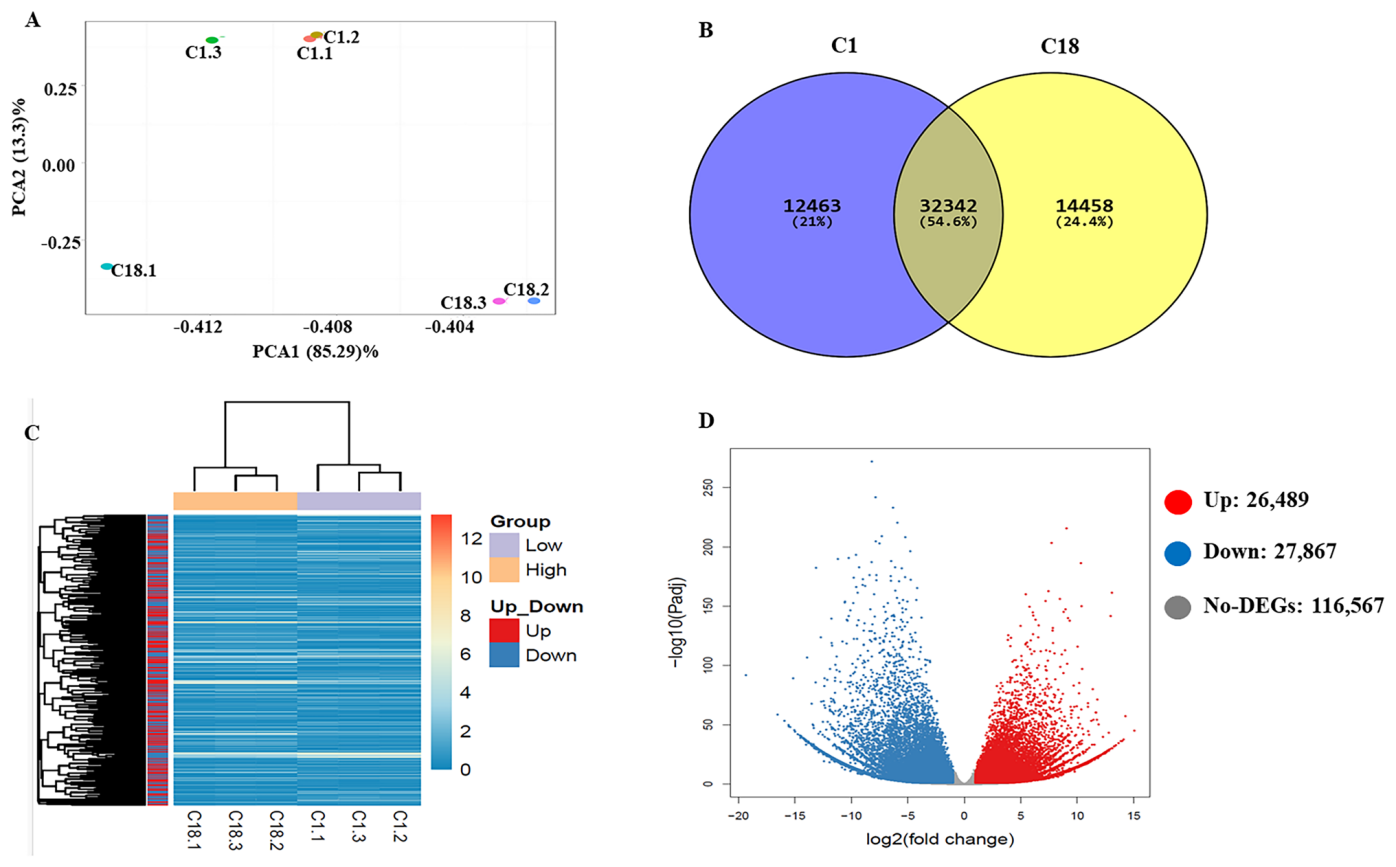
**A** Principal Component Analysis (PCA) of all the expressed transcripts in *C. creticus* C1 and C18 genotypes using FPKM values. **B** Venn diagram depicting the number of genes expressed in both genotypes and uniquely and separately in each genotype. **C** Heatmap of Hierarchical Cluster Analysis based on the differentially expressed transcripts (DEGs) in C1 and C18 genotypes using FPKM values. **D** Volcano diagram for differentially expressed genes in C1 and C18 genotypes. On the right, red represents the genes that are over-expressed, while on the left, blue represents those that are under-expressed in the “high” genotype C18, relative to the “low” C1. In gray, genes are depicted that are not characterized as DEGs

The total transcript number with FPKM ≥ 1 was estimated at 44,805 and 46,800 for the C1 and C18, respectively (Supplementary Table S10; Table S11). The PCA analysis between C1 and C18 explained 98.59% of the total variation in the first two components, revealing that each genotype represented by each replicate was clustered together, showing a clear separation of the “high” from the “low” genotype (Fig. 7A). A total number of 32,342 transcripts were expressed in both genotypes, with 12,463 and 14,458 being uniquely expressed in C1 and C18, respectively (Fig. 7B). Figure 7C displays the significant DEGs, indicating that the biological replicates of each genotype were reproducible (Fig. 7C). DEG analysis between C1 and C18 genotypes with |log2(FC) ≥ 1| and *p* ≤ 0.05 disclosed 24,489 and 27,867 DEGs being up-regulated and down-regulated, respectively (Fig. 7D).

Gene Ontology (GO) enrichment analysis of the DEGs between the C1 and C18 genotypes revealed the most enriched terms classified into BP, CC, and MF. For the up- and down-regulated, the most enriched terms regarding the BP were the cellular process (GO:0009987), and metabolic process (GO:0008152), cell (GO:0005623) and cell part (GO:0044464) were the most enriched terms of CC, and the catalytic activity (GO:0003824) and binding (GO:0005488) were the most enriched terms of MF (Fig. S9A). The DEGs were further analyzed to identify the most significantly functionally enriched pathways, with the majority of DEGs presented in ribosome, while a considerable number are appreciably augmented in the plant-pathogen interaction pathway. In contrast, biosynthesis of diterpenes displayed only a small number of functionally enriched DEGs, which unveil moderate significance (Fig. S9B).

### Analysis of DEGs involved in LRD biosynthetic pathway

To investigate the biosynthetic pathway of specific LRDs i.e., *ent*−3β-ecetoxy-13-epi-manoyl oxide, DEGs involved in LRDs backbone were analyzed in C1 and C18 genotypes. To this end, we utilized the RNA-seq and DEGs therein data to search for candidate genes with high homology to the above genes and transcriptional correlation with the target metabolite. First choice was the previously characterized genes from our laboratory, including *CLS* (HM537017; CL282.Contig6), *LDDS2* (MT666221; CL282.Contig3) and *LAT* (MT666224; CL282.Contig1) (Papanikolaou et al. 2024) and *KS* (MT666222; CL3799.Contig1) (Papanikolaou 2018), followed by an in silico analysis in NCBI non-redundant (Nr) database, which revealed several candidate Class II labdane diterpene synthases (diTPS) including two putative *ent-CLS* (CL282contig13, Unigene9455) and two candidate cytochrome P450 hydroxylases (CL5068contig4, CL17411contig6).

**Fig. 8:**
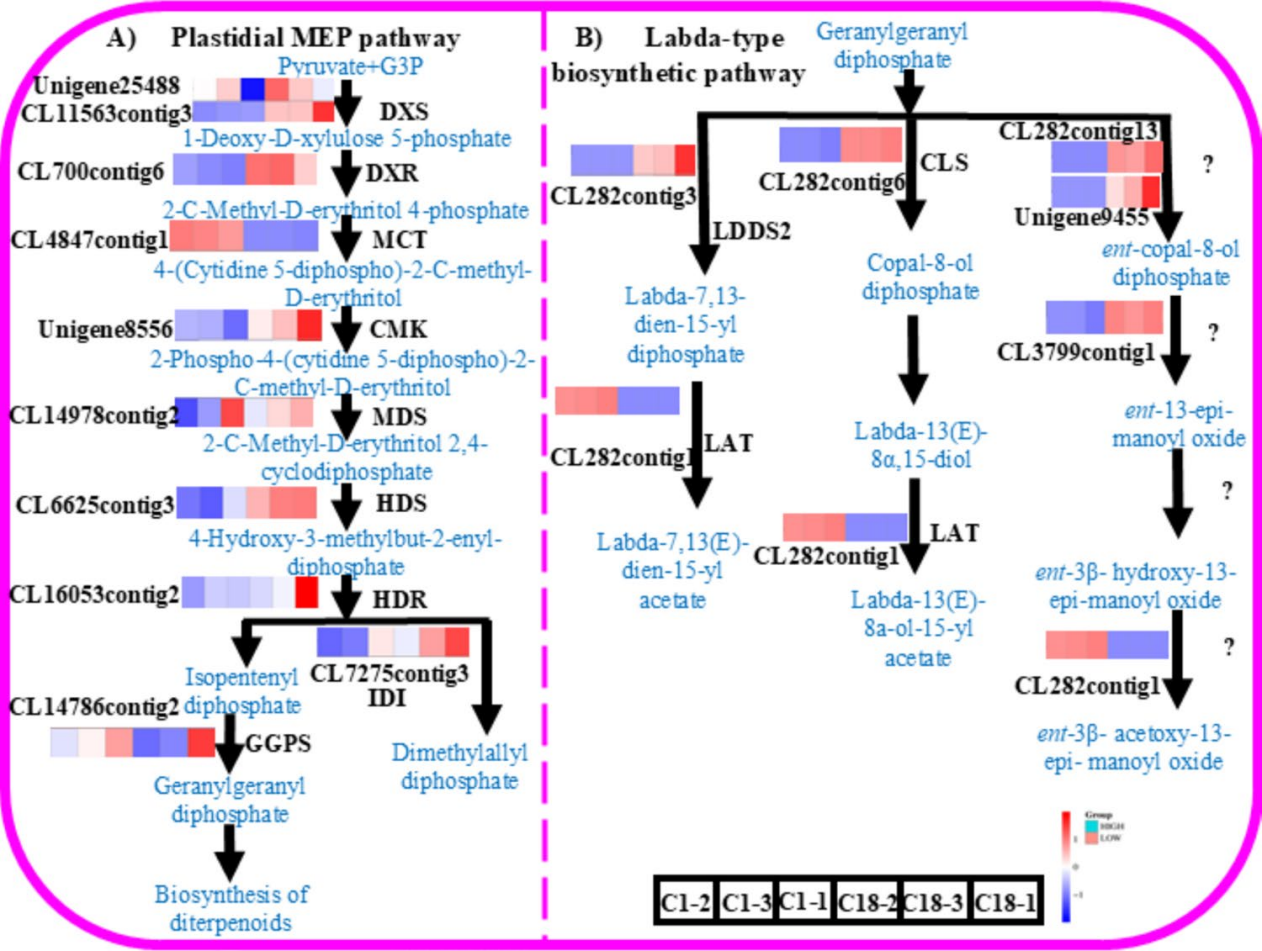
Expression profile of *C. creticus* genes related to the biosynthesis of terpenes using normalized FPKM expression values. **A** Heatmap of the expression of genes involved in the terpenoid backbone biosynthetic pathway for C1 and C18 genotypes in plastids. MEP, 2-C-methyl-D-erythritol 4-phosphate; *DXS* (Unigene25488, CL11563contig3): 1-deoxy-D-xylulose-5-phosphate synthase; *DXR* (CL700contig6): 1-deoxy-D-xylulose-5-phosphate reductoisomerase; *MCT* (CL4847contig1): 2-C-methyl-D-erythritol 4-phosphate cytidylyltransferase; *CMK* (Unigene8556): 4-diphosphocytidyl-2-C-methyl-D-erythritol kinase; *MDS* (CL14978contig2): 2-C-methyl-D-erythritol 2,4-cyclodiphosphate synthase; *HDS* (CL6625contig3): 4-hydroxy-3-methylbut-2-enyl-diphosphate synthase; *HDR* (CL16053contig2): 1-hydroxy-2-methyl-2-(E)-butenyl 4-diphosphate reductase; *IDI* (CL7275contig3): isopentenyl-diphosphate delta-isomerase; *GGPS* (CL14786contig2): geranylgeranyl pyrophosphate synthase. **B** Heatmap of the expression of genes involved in the labdane-related diterpene pathway of *C. creticus*. *CLS* (CL282contig6): copal-8-ol diphosphate hydratase; *LDDS2* (CL282contig3): labda-7,13(E)-dien-15-yl diphosphate synthase; *LAT* (CL828contig1): labdane acetyltransferase; candidate genes, *ent-CPS* (CL282contig13, Unigene9455): *ent*-copalyl diphosphate synthase; CYP p450 hydroxylases (CL5688contig4, CL17411contig6); *KS* (CL3799contig1): kaurene synthase. For the characterized genes, the NCBI accession number is also given: *CLS*-HM537017, *LDDS2*-MT666221, *KS*-MT666222, *LAT*-MT666224

The MEP pathway expressed genes such as *DXS2* gene (CL11563contig3), *DXR* (CL700contig6), *CMK* (Unigene8556), *HDS* (CL6625contig3), and *IDI* (CL7275contig3) were up-regulated in C18 relative to C1. Contrariwise, the following genes, *DXS1* (Unigene25488), *MCT* (CL4847contig1), *MDS* (CL14978contig2), *HDR* (CL16053contig2), and *GGPS* (CL14786contig2) were down-regulated in C18 compared to C1 (Fig. 8A). Genes of the LRDs biosynthesis, including candidate e*nt-CLS* (CL282contig13, Unigene9455), *LDDS2* (*CL282contig3*), *CLS* (CL282contig6), and *kaurene synthase KS* (CL3799contig1), showed increased expression in the C18 genotype. Contrary, the expression of candidate hydroxylases (CL5688contig4, CL17411contig6) as well as *LAT* (CL828contig1), appears to be increased in the low genotype (C1) (Fig. 8B).

Verification of the DEGs analysis was performed by quantitative PCR (qPCR) revealing the expression levels of putative *ent-CLS* CL282contig13 and Unigene9455, *LDDS2* (CL282contig3), *CLS* (CL282contig6), and *kaurene synthase* (CL3799contig1) were significantly higher in the C18 genotype, in contrast to the expression levels of some putative CYPs (CL5068contig4, CL17411contig6) and *LAT* (CL828contig1) increased in the C1 genotype (Fig. S10).

### Transcription factors (TFs) linked to LRDs biosynthesis

**Fig. 9:**
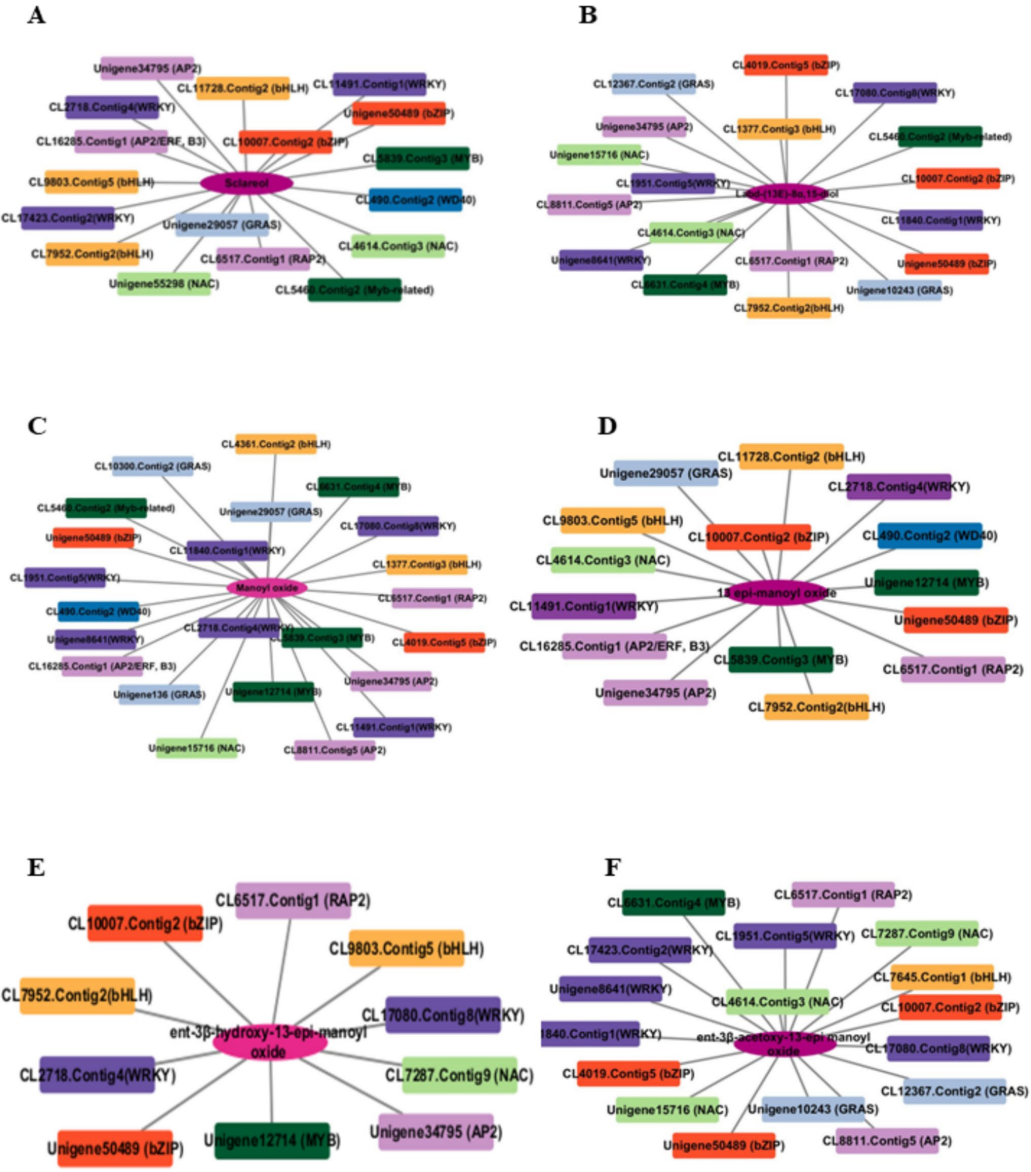
Visual representation of the correlation network between the labdane- related diterpenes. **A** sclareol, **B** labda-(13E)−8α,15-diol, **C** manoyl oxide, **D** 13-epi-manoyl oxide, **E** *ent*−3β-hydroxy-13-epi-manoyl oxide, and **F** *ent-*3β-acetoxy-13-epi-manoyl oxide content (ellipse shape) and the significantly correlated, up-regulated transcription factors (rectangular shape), in the C18-high genotype of *C. creticus*. Each node represents a gene, colored by functional annotation in the Nr base

To delve into the regulatory network of LRDs, correlation analyses were conducted aiming at the identification of potential associations between TFs-amount of specific labdane compounds and TFs-labdane related genes, revealing 68 DEGs belonging to eight TF families (WRKY, MYB, AP2, bHLH, bZIP, NAC, WD40, GRAS) that were significantly up-regulated in the C18 genotype. Notably, the *RAP2* (CL6517contig1), *bZIP* (Unigene50489) were associated with all the labdane compounds in this study (Fig. 9). Interestingly, no association was detected between the WD40 TF family and labda-(13E)−8α,15-diol, *ent*−3β-hydroxy-13-epi-manoyl oxide, and *ent-*3β-acetoxy-13-epi-manoyl oxide (Fig. 9B, E, F), as well as the MYB and GRAS family with manoyl oxide and *ent-*3β-hydroxy-13-epi-manoyl oxide, respectively. The *bZIP* (CL10007contig2) was the only transcription factor significantly correlated with the 13-epi-manoyl oxide, *ent-*3β-hydroxy-13-epi-manoyl oxide, and *ent*−3β-acetoxy-13-epi-manoyl oxide (Fig. 9D–F).

Regarding the construction of the correlation network delineating the biosynthesis of the hydroxy-derivative of 13-epi-manoyl oxide, different and distinct patterns were observed for the two candidate CYPs. Specifically, the CL5068contig4 displayed a negative correlation with all the investigated TFs, whereas the other candidate CYP CL17411contig6 presented correlation trends with the examined TFs, but these relationships were below the statistical threshold of *r* ≥ *0.95* and *p* < *0.05* (data not shown). In contrast, the diTPSII CL282contig13 exhibited strong positive correlation with the *AP2* (CL16285conti1, Unigene34795, CL6517contig1), *WRKY* (CL11491contig1, CL17423contig2, CL2718contig4), *bHLH* (CL11728contig2, CL7952contig2, CL9803contig5), *bZIP* (CL10007contig2), *NAC* (CL4614contig3), *WD40* (CL490contig2), *GRAS* (Unigene29057) and *MYB* (CL5839contig3, CL5460contig2) family (Fig. S11A). On the other hand, the kaurene synthase CL3799contig1 was correlated with the *AP2* (CL16285conti1, Unigene34795, CL8811contig4, CL8811contig5), *WRKY* (CL17080contig3, CL17423contig2, CL2718contig4, CL11840contig1), *bHLH* (CL1377contig3), *NAC* (CL7287contig9, Unigene15716), *WD40* (CL490contig2), *GRAS* (Unigene29057, CL10300contig2, Unigene136) and *MYB* (CL6631contig4, CL5839contig3, Unigene12714) family. It is evident that the CL3799contig1 had no correlation with the bZIP family (Fig. S11B). Lastly, correlation analysis between labdane compounds and their putative biosynthetic genes showed that diTPSII CL282contig13 was significantly positively correlated with 13-epi-manoyl oxide and sclareol, whereas Unigene9455 was significantly correlated with 13-epi-manoyl oxide, sclareol, and *ent*−3β-hydroxy-13-epi-manoyl oxide (Fig. S11C). The characterized diTPSII and diTPSI from our lab (Falara et al. 2010; Papanikolaou et al. 2024) (*CLS,* CL282contig6; *KS,* CL3799contig1) had a significantly strong correlation with the 13-epi-manoyl oxide, and *ent*−3β-hydroxy-13-epi-manoyl oxide. However, two CYPs (CL5068contig4, CL17411contig6) that might participate in the biosynthesis of the *ent-*3β-hydroxy-13-epi-manoyl oxide, had no significant correlation with any of the labdane compounds (Fig. S11C).

### Identification of variations in *C. creticus* transcriptome

SNP and InDel variants were identified using the RNA-seq data. The filtered reads were aligned to the *de-novo* transcriptome assembly, revealing 347,213 SNPs, 15,386 insertions, and 24,750 deletions. The genomic distribution of these variants illustrated that the majority of both SNPs (40.4%) and InDels (55.37%) were located in the 3′ and 5′ UTR regions (Supplementary Table S12). Among all SNPs, 81,539 were missense (non-synonymous) and 105,103 were synonymous (silent) mutations. Based on their predicted functional impact, 0.34% of SNPs were classified as high impact, 25.59% as low impact, 18.03% as moderate impact, and 56.04% as modifiers.

For the 40,136 InDels, 10.47% were predicted to have a high impact, while 5.81% and 83.71% were categorized as moderate and modifier impact, respectively. We also examined SNP and InDel variants specifically within the coding regions of the target genes [*CLS, KS, LAT, CYP*]. Only one in-frame deletion (9 nucleotides) was detected in the *KS* coding region (CL3799contig1) (Supplementary Table S12). Several SNPs were identified within this gene set, including multiple homozygous missense variants resulting in amino acid substitutions (Supplementary Table S12). These variants highlight clear sequence divergence between C1 and C18 across several pathway genes. Interestingly, most of the detected mutations in *KS, LAT*, and the putative *CYP* genes are missense or synonymous, suggesting moderate effects on protein function rather than disruptive changes.

## Discussion

The present study provides a comprehensive genetic, chemical, and transcriptomic characterization of *C. creticus* genotypes from various populations and geographical regions, offering insights into the factors affecting its diversity and accumulation of LRDs. Previous genetic studies in *C. creticus* have mainly focused on the classification of the species and subspecies using molecular markers (Guzmán and Vargas 2005; Fernández-Mazuecos and Vargas 2011; Lukas et al. 2021). In contrast, our study investigates the genetic differentiation within *C. creticus* subsp *creticus*, providing a thorough investigation of intraspecific diversity.

The study of the 91 *C. creticus* genotypes using ISSR markers revealed notable population-level polymorphism and a substantial degree of genetic differentiation among the seven regions (Table 1; Table 2). These results contrast with earlier reports by Falchi et al. (2009) and Paolini et al. (2009) who observed lower inter-population variation. Such differences may not only be attributed to the distinct starting material used in those studies, including different subspecies (*creticus* and *eriocephalus* of *C. creticus)*, as well as the influence of geographic and environmental factors on population structure. The observed differentiation in our dataset also suggests limited gene flow, likely driven by the geographical isolation of the understudied populations (Supplementary Table S1). Nevertheless, the majority of genetic variation was detected within populations, indicating a rich local gene pool that represents valuable material for breeding programs aiming to enhance desirable traits under specific environmental conditions.

At the population level, the genotypes of Floria (F) exhibited the highest polymorphism, Shannon’s index, and Nei’s gene diversity (Table 1), indicating a high degree of genetic diversity and highlighting its importance for both in situ and ex situ conservation programs. In contrast, the population of Agrinio (Ag) showed the lowest diversity values, potentially reflecting a bottleneck or increased genetic drift due to isolation.

Clustering analysis (UPGMA, PCoA, STRUCTURE) consistently demonstrated that the genetic structure of *C. creticus* populations is primarily shaped by the geographic origin. The populations of Chalkidiki and Sises formed distinct and homogenous clusters, whereas Akrotiri population stands out as the most heterogeneous, with STRUCTURE analysis revealing moderate admixture levels (Fig. 1; Fig. 2; Fig. S3; Fig. S4). Interestingly, UPGMA divided the populations into two major clusters, while the PCoA and STRUCTURE identified three clusters. This is not contradictory but complementary, as UPGMA captures deeper historical splits, while STRUCTURE and PCoA resolve more recent or complex patterns. Similar observations have been reported in other species, such as *Camellia,* where different clustering approaches identified distinct aspects of population history (Yan et al. 2022).

ISSR markers associated with LRD content indicate that these particular markers can serve as a possible tool for screening *C. creticus* germplasm for high-LRD genotypes. Such marker-trait relations represent a valuable resource for breeding and have been successfully applied in other species, such as cherry (Ganopoulos et al. 2011), and mastic trees (Kostas et al. 2021). Labdane-related diterpenes have been consistently reported as characteristic constituents of *C. creticus*, yet the quantitative and qualitative variation observed across populations and tissues highlights the chemical diversity of this species. Demetzos et al. (1997) first identified manoyl oxide and 13-epi-manoyl oxide as major diterpenes in the essential oil of *C. creticus* from Sises, while Falara et al. (2010) demonstrated that trichomes of young leaves are a specialized site for their biosynthesis. Later, Demetzos et al. (2002) extended this work by analyzing 25 populations across Crete, showing that essential oil profiles could be grouped into three chemotypes, reflecting both genetic and geographical differentiation. Anastasaki et al. (1999) quantified diterpenes in dried *C. creticus* leaves, reporting 13-epi-manoyl oxide at 2.48 μg/g, while the present dataset, which analyzed fresh leaves at early developmental stages, yielded markedly higher average concentrations of 93.44 μg/g (29 genotypes from Sises). Similarly, 13-epi-manoyl oxide was previously detected at 2.61 μg/g (Anastasaki et al. 1999), while the present data show 29.34 μg/g. Other diterpenes, such as labda-7,13-dien-15-ol and its acetylated derivative, were also quantified by Anastasaki et al. (1999) who reported 14.9 μg/g and 32.6 μg/g in *C. creticus* leaves, versus 37.19 μg/g 48.58 μg/g, in the present analysis of fresh leaves. In comparison, *C. creticus* showed lower values in Anastasaki et al. (1999) while higher concentrations were detected here, underscoring strong population-level differentiation.

A point of novelty in the present dataset is the magnitude of variation among genotypes within a single population, with individuals such as genotype C6 from Chalkidiki producing 323.9 µg/g leaf of 13-epi-manoyl oxide and genotype C18 from Chalkidiki yielding 231.0 µg/g leaf of *ent-*3β-acetoxy-13-epi-manoyl oxide, levels rarely reported in absolute terms in the literature. This indicates that selecting suitable productive genotypes for cultivation could significantly increase the yield of plants in bioactive substances. Importantly, LRDs were also found in ladano resin at even higher levels (Anastasaki et al. 1999), indicating that both tissue type and extraction method were significantly affected by the diterpene yields. Taken together, these comparisons reinforce earlier conclusions that *C. creticus* exhibits substantial intraspecific chemical polymorphism (Demetzos et al. 2002), while the absolute quantification presented here demonstrates that fresh leaf tissues of certain genotypes, particularly from Chalkidiki, accumulate labdane diterpenes at concentrations not commonly reported in previous studies.

Several studies have applied multiple regression or similar association analyses to link ISSR (or ISSR + RAPD) markers with chemical or yield traits, showing results that are broadly comparable to the MRA on *C. creticus*. In endemic taxa, Rajčević et al. (2022) combined ISSR and phytochemical profiling and observed congruence between genetic groups and metabolite accumulation, much like the positive and negative correlations reported among specific UBC bands and diterpene yield in *C. creticus*. Targeted studies in *Capparis spinosa*, (Grimalt et al. 2022) showed that ISSR genetic profiles correspond closely with chemical composition and antioxidant activity of flower buds. These findings indicate that molecular fingerprints can serve as markers for metabolite levels, while similar findings emerge in the present work, with the identification of strong effect markers, such as UBC808_800_ (*β* = 0.332, *t* = 4.671).

Specialized metabolites are natural products of pharmaceutical plants, which are responsible for their pharmacological properties (Bozoglan et al. 2025). Among them, *C. creticus*, valued in traditional medicine, is recently gaining attention for its potent antiviral activities against Sars-Cov-2 (Bozoglan et al. 2025), properties attributed to specialized secondary metabolites consisting of LRDs and flavonoids (Papaefthimiou et al. 2014; Bozoglan et al. 2025). Structural complexity of LRDs gives rise to a wide range of bioactivities, valuable not only in plant defense but in development of novel agrochemicals and pharmaceuticals (Bian et al. 2024). To comprehend the mechanisms behind the accumulation of LRDs, a transcriptomic approach on specific leaf tissue (S1 developmental stage) was carried out, investigating whether the compounds variation in two selected genotypes (C1 low in LRDs, C18 high in LRDs) was resulted by different expression profiles of the LRD biosynthetic genes and transcription factors, or it was caused by the interplay between plants and their growing environment.

The enrichment analysis demonstrated a considerable increase in diterpenoid biosynthetic-related genes in the C18 genotype (Fig. S9B), implying a further exploration of the expression patterns in the LRD biosynthetic pathway. Differential expression analysis revealed an up-regulation of the two putative diTPSII (*ent-CLS: *CL282contig13, Unigene9455) and *kaurene synthase* gene (*KS: *CL3799contig1) in C18, leading to the biosynthesis of *ent-*manoyl oxide. Moreover, the *copal-8-ol diphosphate synthase* (*CLS: *CL282contig6) and *labda-7,13(E)-dien-15-yl diphosphate synthase* (*LDDS2: *CL282contig3), the precursors of the labda-(13E)−8α,15-diol and labda-7,13-dien-15-yl diphosphate compounds, were also up-regulated, supporting an increased flux toward labdane intermediates (Fig. 8B). These findings align with the chemical analysis, which revealed genotype-specific accumulation of specific LRDs in the C18 compared to C1 (Fig. S5). Because all plants were grown under identical environmental conditions, the differences likely reflect inherent genetic and transcriptional features, consistent with patterns reported in *S. officinalis* and *S. rosmarinus*, where the higher expression of the structural genes in the carnosic acid biosynthetic pathway is genotype-driven (Božić et al. 2015; Scheler et al. 2016; Ali et al. 2017; Ntoanidou et al. 2024, 2026).

In parallel with the transcriptomic analysis, the variant calling results provided additional insight into the molecular differentiation between C1 and C18. Although both genotypes shared most coding sequences, a substantial number of SNPs and InDels were identified across the transcriptome, with UTRs representing the most variant-dense regions. Interestingly, only a single in-frame deletion was detected within the coding region of *KS*, and most mutations in LRD-related genes were synonymous or moderate-impact missense variants. This suggests that the pronounced differences in LRD accumulation between C1 and C18 are primarily driven by transcriptional regulation rather than major structural alterations in the coding sequences of key biosynthetic genes.

Quantitative PCR confirmed the transcriptomic analysis of all the understudy genes showing an increased relative expression of the two putative diTPSII (*ent-CLS: *CL282contig13, Unigene9455), *KS* (CL3799contig1), *CLS* (C*L282contig6*), *LDDS2* (CL282contig3) in the C18 genotype (Fig. S10; Supplementary Table S11), results that concede with the ones reported by Ntoanidou et al. (2024) where the genotypes with high accumulation of carnosic acid had higher relative expression level of *CPS2* (Ro209030) in *S. rosmarinus*. In contrast, the relative expression of some CYPs (CL5068contig4, CL17411contig6) and *LAT* (CL828contig1) increased in the C1 genotype (Fig. S10; Supplementary Table S11). Although the transcript levels of the *LAT* gene were elevated in the C1 genotype (RNA-seq, qPCR), the amount of the corresponding compounds (e.g., *ent*−3β-acetoxy-13-epi-manoyl oxide, Labda-7,13-dien-15-yl acetate) was elevated in the C18 genotype. This divergence of direct correlation between transcript abundance and compound content is a known phenomenon in plant specialized metabolism, where the transcriptional up-regulation does not always interpret into metabolic flux. For example, the production of specific ester compounds may be governed by substrate availability rather than enzyme abundance, as the maximum compound production did not correlate with the peak of enzyme activity suggesting that precursor supply serves as the primary limiting factor (Shalit et al. 2003), suggesting a similar way of regulation in *C. creticus*. These findings highlight that in specialized metabolism the production of specialized compounds is a complex process integrating upstream precursors synthesis, availability, and quantity, along with protein regulation.

Aiming to gain insight into how to optimize the biosynthetic pathway of LRDs by enhancing the expression of key biosynthetic genes to develop varieties with enhanced traits, TFs were searched in the *C. creticus* transcriptome, as TFs are known to regulate alterations in compound content by operating as molecular switches binding to specific DNA sequences of promoter regions. This research in the up-regulated genes revealed 68 DEGs belonging to eight TF families (WRKY, MYB, AP2, bHLH, bZIP, NAC, WD40, GRAS), which are known for their involvement in the regulation of diterpene production (Junze et al. 2022). Particularly, the CL9803contig5 and CL1377contig3 belonging to the bHLH family were strongly and significantly correlated with the putative diTPSII CL282contig13 (*ent-CLS*), 13-epi-manoyl oxide, *ent-*3β-hydroxy-13-epi-manoyl oxide, and *KS* (CL3799contig1), manoyl oxide, respectively (Fig. 9; Fig. S11). Indeed, our analysis is supported by a work in rice where the binding of TF *OsbHLH025* to the N-Box motif of the promoter region of the *OsCPS2* (producing *ent-CPP*) biosynthetic gene enhanced the production of the LRD phytoalexin (Bian et al. 2024). Above all, the overexpression of *OsbHLH025-OX* enhanced the production of diterpenoids even under normal conditions (Yamamura et al. 2015; Bian et al. 2024).

The CL10007contig2, a member of the bZIP family, exhibited strong association with the putative diTPSII CL282contig13 (*ent-CLS*), 13-epi-manoyl oxide, *ent-*3β-hydroxy-13-epi-manoyl oxide, and *ent*−3β-acetoxy-13-epi-manoyl oxide, indicating a regulatory relationship (Fig. 9; Fig. S11). Similar to these findings, Okada et al. (2009) reported that in the *OsTGAP*1 (bZIP factor) overexpressing lines, the expression level of the *OsKSL4*, *OsKSL7* and *OsDXS3* was up-regulated after the induction with chitin oligosaccharide elicitor, leading to an increased amount of phytoalexins (Bian et al. 2024). The WRKY transcription factors CL2718contig4, CL11491contig1 and CL11840contig1 were strongly associated with the putative diTPSII CL282contig13 (*ent-CLS*), *KS* (CL3799contig1), manoyl oxide, 13-epi-manoyl oxide, *ent-3β-hydroxy-13-epi-manoyl* oxide and *ent-*3β-acetoxy-13-epi-manoyl oxide suggesting a crucial role for these TFs in LRD biosynthesis (Fig. 9; Fig. S11), an observation that is coherent with the induction of diterpenoid phytoalexins (LRDs) in transgenic rice overexpressing the TF *OsWRKY45-OX* upon infection with *M. oryzae* (Akagi et al. 2014; Bian et al. 2024). Furthermore, the detected correlation between specific NAC, GRAS, MYB, WD40, ERF TFs and structural genes (CL282contig13, CL3799contig1) and their corresponding compounds (manoyl oxide, 13-epi-manoyl oxide, *ent-*3β-hydroxy-13-epi-manoyl oxide and *ent*−3β-acetoxy-13-epi-manoyl oxide) (Fig. 9; Fig. S11), indicates a vital regulatory involvement of these TFs in the production of LRDs. Nonetheless, this finding presents an initial investigation, compelling further experimental validation to establish a conclusion regarding their regulatory mechanisms.

**Fig. 10:**
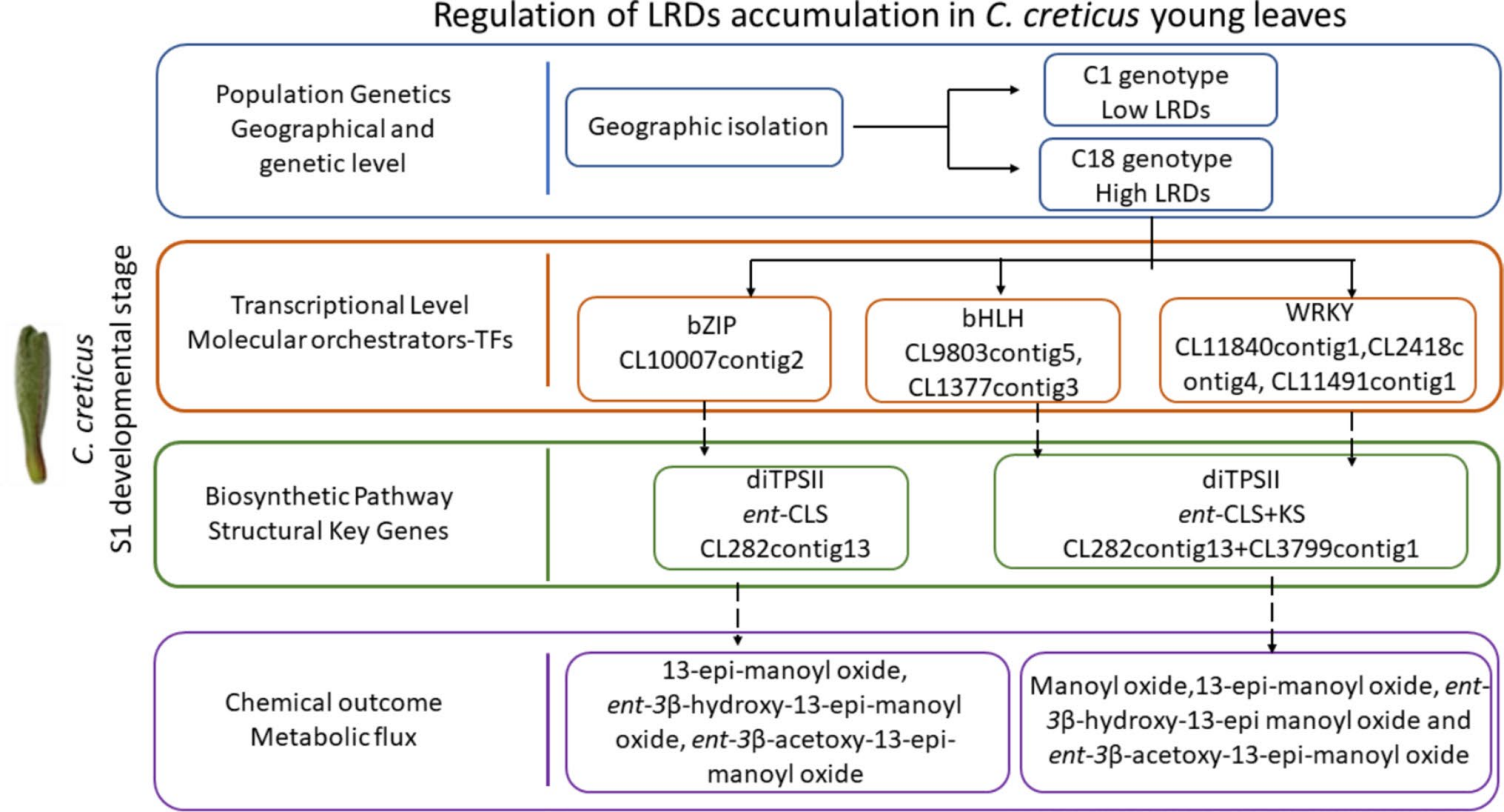
Proposed transcriptional and metabolic regulatory model governing *C. creticus* labdane-related diterpenes (LRDs) biosynthesis in leaves of S1 developmental stage. The scheme incorporates genetic, transcriptomic, and chemical data illustrate the differences in LRDs accumulation between low-producing (C1) and high-producing (C18) genotypes. *ent*-*CLS*, *ent*-copalyl diphosphate synthase; *KS*, kaurene synthase

To combine these multi-faceted results, we proposed a regulatory model governing the biosynthesis of LRDs in *C. creticus* young leaves, by linking the gap between genetic variation transcriptional regulation and metabolic phenotypes (Fig. 10). As the variant calling analysis in both genotypes revealed that most mutations in LRD-related genes were synonymous or moderate-impact missense variants suggesting that the substantial divergence in LRD accumulation is primarily driven by transcriptional regulation rather than structural changes in biosynthetic enzymes. The proposed model highlights the role of specific TF orchestrators particularly within the bHLH, bZIP and WRKY families, exhibiting a strong correlation with the LRDs’ key structural genes such as *ent*-*CLS* and *KS*. By binding to specific regions in the promoter sequences of these genes, these TFs likely coordinate the transcriptional up-regulation observed in the high-producing LRDs genotype (C18) (Fig. 10).

Conclusively, this study by focusing on the integration of genetic, chemical, and transcriptomic analyses of *C. creticus* facilitated the selection of the richest genotypes in LRDs. By utilizing ISSR markers, the genetic analysis of the *C. creticus* genotypes effectively delineated the genetic landscape, showing a strong clustering according to their geographical origin that can be exploited in breeding programs by crossing individuals from highly distinct populations to generate novel combinations of desirable traits. Chemical analyses of the 91 *C. creticus* genotypes showed differentiation in the LRDs accumulation. Based on the productivity of *ent*−3β-hydroxy-13-epi-manoyl oxide and *ent*−3β-acetoxy-13-epi-manoyl oxide, two genotypes, C1 and C18, low and high, respectively, were selected for RNA-sequencing and transcriptomic analysis, revealing a complex regulatory network for LRDs biosynthesis in the *C. creticus* transcriptome, exposing various TF families that may be orchestrating the expression of key biosynthetic genes (Fig. 10). The identified strong correlation between TFs and structural genes, coupled with the observed specificity for particular TF-compound associations, underscores the complicated control mechanisms that influence the plant’s specialized metabolic profile (Fig. 10). Future studies employing overexpression or knockdown approaches will be necessary to fully characterize the role of these TFs in regulating LRDs.

## Supplementary Information

Below is the link to the electronic supplementary material.

Supplementary file1 (XLSX 15 KB)

Supplementary file2 (XLSX 11 KB)

Supplementary file3 (XLSX 78 KB)

Supplementary file4 (XLSX 12 KB)

Supplementary file5 (XLSX 12 KB)

Supplementary file6 (XLSX 32 KB)

Supplementary file7 (XLSX 11 KB)

Supplementary file8 (XLSX 11 KB)

Supplementary file9 (XLSX 11 KB)

Supplementary file10 (XLSX 29931 KB)

Supplementary file11 (XLSX 34637 KB)

Supplementary file12 (XLSX 18 KB)

Supplementary file13 (DOCX 4870 KB)

## Data Availability

The experimental data that support the findings of this study are available in the Supplemental Materials. In addition, any additional requested data of this article will be shared on request to the corresponding author.

## Abbreviations

DEG: Differentially expressed gene
ISSR: Inter simple sequence repeats
LAT: Labdane acetyltransferase
LRDs: Labdane-related diterpenes
SNP: Single nucleotide polymorphism
TF: Transcription factor
TPS: Terpene synthase
