# Supplementary material for: Harnessing genetic, chemical, and transcriptomic analyses guided the selection of Cistus creticus subsp. creticus genotypes rich in labdane-type diterpenes
Source: Planta. 2026 Jul 15;264(2):53. doi: 10.1007/s00425-026-05072-1 (PMC13372853; doi:10.1007/s00425-026-05072-1)
Supplement: Supplementary file 13 — Supplementary file13 (DOCX 4870 KB) [file 425_2026_5072_MOESM13_ESM.docx]

**Supplementary Information**

**Harnessing genetic, chemical, and transcriptomic analyses guided the selection of *Cistus*** ***creticus* subsp. *creticus* genotypes rich in labdane-type diterpenes**

**Fig S1.** Separation of PCR amplification products generated with primer UBC 818 using genomic DNA from 18 genotypes following agarose gel electrophoresis. A 1 kb DNA ladder was loaded at both edges of the gel. Image were converted to grayscale and inverted (negative) for improved band visualization.

**Fig. S2** Mass spectra of the most abundant labdane-type diterpene peaks and their comparison with mass fragments from Papanikolaou et al. (2024) and the similarity identified with the FFNSC 2 GC-MS library.

**Fig. S3** Principal Coordinates Analysis (PCoA) derived from bands amplified by 11 ISSR primers among the 7 populations and the 91 genotypes of *C. creticus* from Greece. Populations are distinguished by colored symbols, while genotypes by the letters next to each symbol. The *C. creticus* individuals grouped by PCoA as genotypes from Sises, in the blue circle, genotypes of Chalkidiki, in the red circle, and the genotypes from the other locations, in green circle.

**Fig. S4** Geographical distribution (map of Greece) of the seven populations of C. creticus and STRUCTURE-based genetic relationships of the corresponding genotypes. Each point represents a population location, and the colored markers charts up to each point, indicate the proportion of each genotype assigned to the respective three (ΔK=3) STRUCTURE clusters

**Fig. S5** Concentrations of sclareol oxide, manoyl oxide, 13-epi-manoyl oxide, 13(16),14-labdien-8-ol, abienol, sclareol, labda-7,13-dien-15-ol, *ent-*3β-hydroxy-13-epi-manoyl oxide, *ent-*3b-acetoxy-13-epi-manoyl oxide, labda-7,13(E)-dien-15-yl acetate, labda-(13E)-8α,15-diol and labda-(13E)-8α,15-yl acetate in μg/g fresh young leaves of *C. creticus* growing in Experimental Field: (A) Allocation of the 91 genotypes (n=3, from sampling in three different plants-clones of the same genotype) of *C. creticus*. The 40 main genotypes are presented, the 20 with the highest rates, the 20 with the lowest rates and the average (AV) of all 91 genotypes. (B) The 7 populations (C: Chalkidiki, S: Sises, A: Akrotiri, F: Floria, Ch: Chios, Ag: Agrinio and M: Manoliopoulo) of *C. creticus* in descending order. Same letters indicate statistical similarity between rates (Duncan criterion, p <0.05).

**Fig. S6** Heatmap of 12 labdane-type diterpenes detected in young leaves from 91 *C. creticus* genotypes versus their places of origin. Plants were growing in Aristotle University of Thessaloniki Experimental field (Thermi area), sampled from young leaves. Genotypes from Chalkidiki exhibited the highest values where those from Manoliopoulo and Agrinio had the lowest content.

**Fig. S7** Concentration of total labdane-type diterpenes in μg/g fresh young leaves of *C. creticus* growing in outdoor plant collection: (A) Allocation of the 91 genotypes (n=3, from sampling in three different plants-clones of the same genotype) of *C. creticus*. The main 40 genotypes are presented, the 20 with the highest rates, the 20 with the lowest rates and the average (AV) of all 91 genotypes. (B) The 9 populations of *C. creticus* in descending order. Codes indicate the origin of the genotypes C: Chalkidiki, S: Sises, A: Akrotiri, F: Floria, Ch: Chios, Ag: Agrinio and M: Manoliopoulo. Same letters indicate statistical similarity between rates (Duncan criterion, *p* <0.05).

**Fig. S8** Scatterplot showing the positive correlation of Regression Standardized Predicted Value and the total diterpenes production in *C. creticus* leaves, as determined by multiple regression analysis (MRA).

**Fig. S9** A) Classification of the number of over- and under-expressed DEGs of C18 according to Gene Ontology. B) Functional enrichment of DEGs in the various biochemical pathways based on KEGG. The size of the circles represents the number of genes proportionally. The color refers to the q-value, (the lower is darker shade of blue), the more significant the enrichment. On the horizontal axis, the enrichment factor resulting from the quotient of the number of DEGs with the total number of genes, is another criterion of significance of functional enrichment. The higher its value, the more significant the enrichment.

**Fig. S10** Expression of key enzymes in the biosynthetic pathway of labdane diterpenes in leaves of *C. creticus*. Leaves of C1 (low) and C18 (high) genotypes that were used in this assay were at S1 developmental stage. *C. creticus* actin and elongation factor was used as internal controls, and expression of each gene was normalized to the average of *C. creticus* actin and elongation factor using the 2^-ΔCt^ method. Bars represent the mean of two biological replicates per genotype ± SE. Statistical significance was assessed with Student test at *α=0.05*. Abbreviations*: CLS: copal-8-ol diphosphate synthase; KS: kaurene synthase; LDDS2: labd-7,13(E)-dien-15-yl diphosphate; LAT: labdane acetyltransferase.*

**Fig. S11** Network analysis of Transcription Factors (rectangular shape) and genes (ellipse shape) of A) putative *diTPSII CL282contig13* and B) *kaurene synthase CL3799contig1* in the biosynthesis of the hydroxy-derivative of *ent*-13-epi-manoyl oxide, C) correlation analysis labdane diterpenes’ content and genes related to their biosynthesis. Significantly correlated TF–gene pairs (r≥ 0.95 and p <0.05) were selected to construct the transcriptional regulatory network.

**Supplementary file 1**. Geographical coordinates of the collected 91 genotypes from 7 regions of Greece (populations) of *C. creticus* used to create the experimental collection in Aristotle University of Thessaloniki Farm.

**Supplementary file 2**. Presentation of 11 primers of the ISSRs molecular markers selected for the genetic analysis of the 91 *C. creticus* genotypes. The minimum and maximum size (bp) of bands, annealing temperature, amplified and polymorphic bands, percentages of polymorphism and PIC values is also given.

**Supplementary file 3.** Qualitative determination of the chemical constituents of *C. creticus* leaves. RT denotes the retention time at the peak maximum (s); Actual RI and Expected RI correspond to the experimentally determined and literature Kováts retention indices obtained by GC–MS; m/z indicates the mass-to-charge ratio of the characteristic ions

**Supplementary file 4.** Identification and relative quantification of labdane-type diterpenoids in *C. creticus* leaf extracts. Compounds were identified based on their retention times (RT), experimental retention indices (RI), and comparison with literature values. Relative abundance is expressed as the normalized area relative to an internal standard (IS). S1 and S2 represent the two developmental stages analyzed.

**Supplementary file 5**. Primer sequences of the 8 genes involved in the biosynthesis of labdane diterpenes used for qRT-PCR for the plant species *C. creticus*.

**Supplementary file 6.** Mean of concentrations (n=3, ± standard error) of the labdane type diterpenes in μg/g fresh young leaves of *C. creticus* growing in outdoor plant collection.

**Supplementary file 7**. Statistical data of the reads of the two *C. creticus* genotypes from RNA sequencing, after processing to remove low-quality reads and adapters.

**Supplementary file 8**. Qualitative data of predicted coding regions (CDS) for genotypes C1 and C18 of the species: Qualitative characteristics of transcriptome assembly for the C18/high and C1/low genotypes of *C. creticus*.

**Supplementary file 9**. Qualitative characteristics of Unigenes for the genotypes C1 (low yield) and C18 (high yield) of *C. creticus* for each biological replicate, but also for the single list of Unigenes.

**Supplementary file 10**. FPKM values of the two genotypes.

**Supplementary file 11**. Differentially expressed genes (DEGs) of the two *C. creticus* genotypes.

**Supplementary file 12**. Detailed table of SNPs/InDels of genotypes C1 and C18, compared to de-novo transcriptome assembly. The table shows the genes of interest, the location of the change, the type of effect and whether the genotypes are homozygous (0/0, 1/1) orheterozygous (0/1).

**Supplementary Information, Figs S**

| 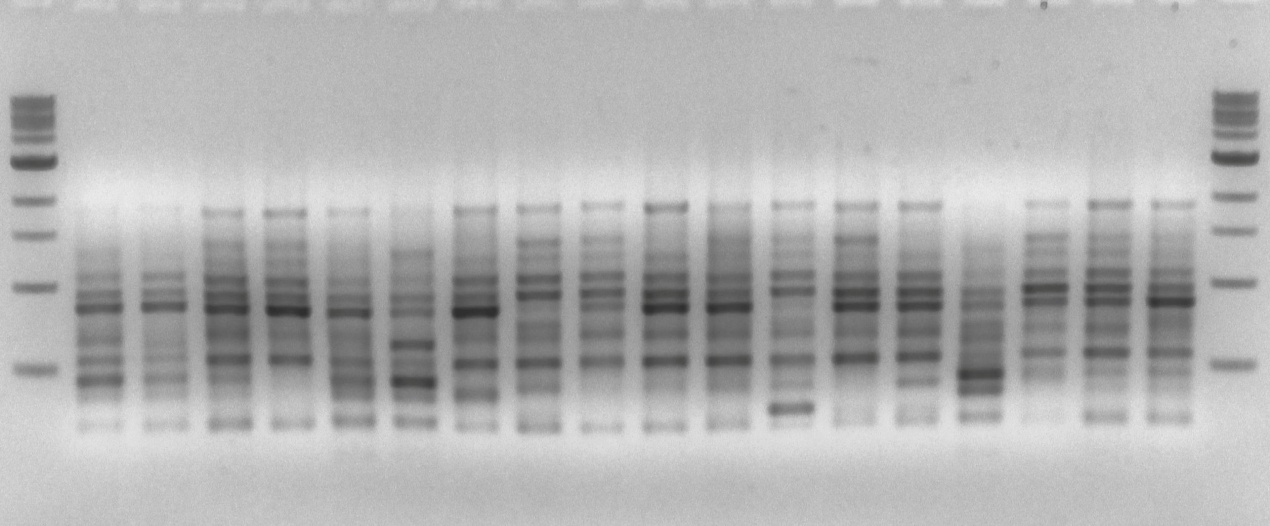 |
| --- |

**Fig. S1** Separation of PCR amplification products generated with primer UBC 818 using genomic DNA from 18 genotypes following agarose gel electrophoresis. A 1 kb DNA ladder was loaded at both edges of the gel. Image were converted to grayscale and inverted (negative) for improved band visualization.


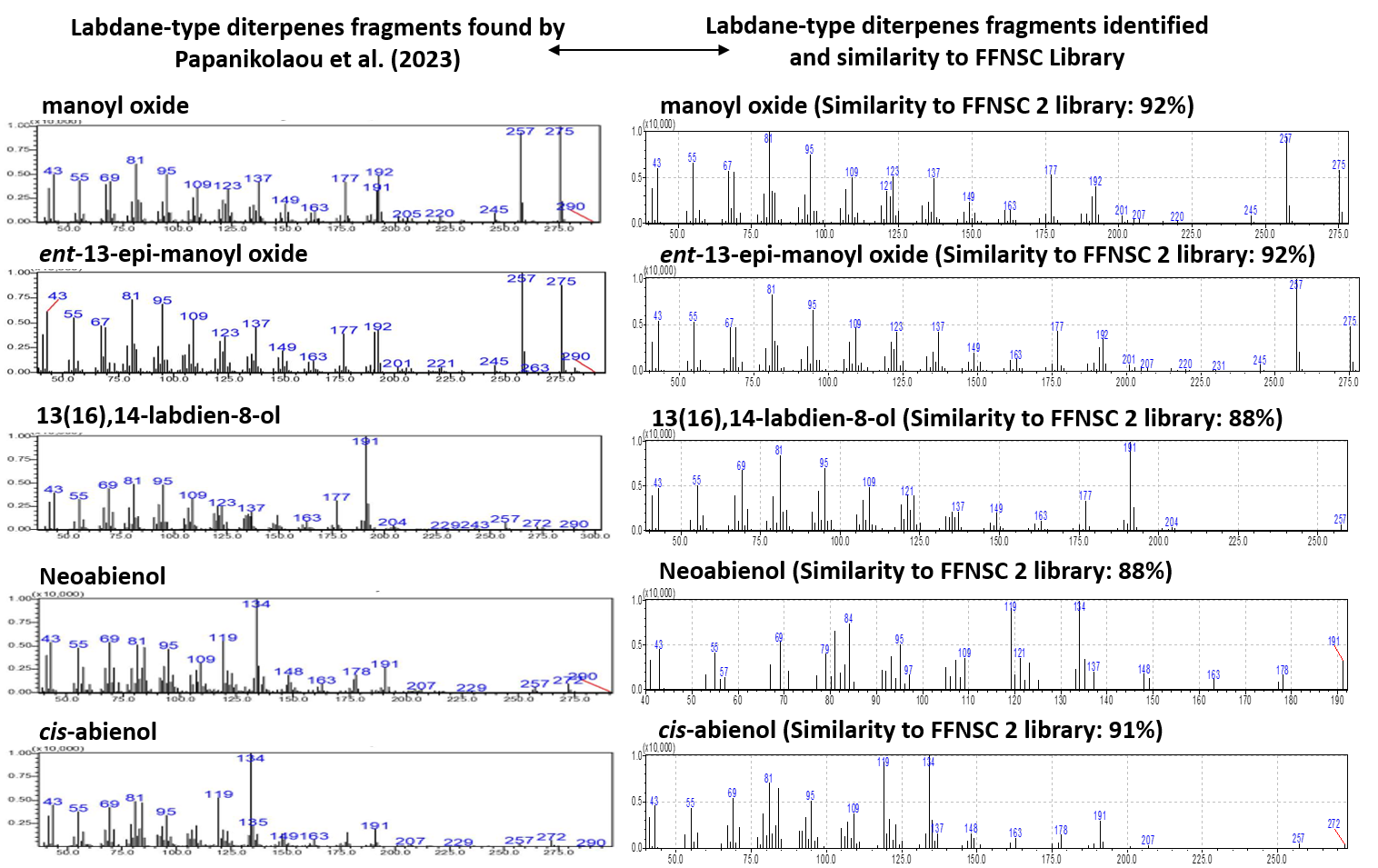


**Fig. S2** Mass spectra of the most abundant labdane-type diterpene peaks and their comparison with mass fragments from Papanikolaou et al. (2024) and the similarity identified with the FFNSC 2 GC-MS library.


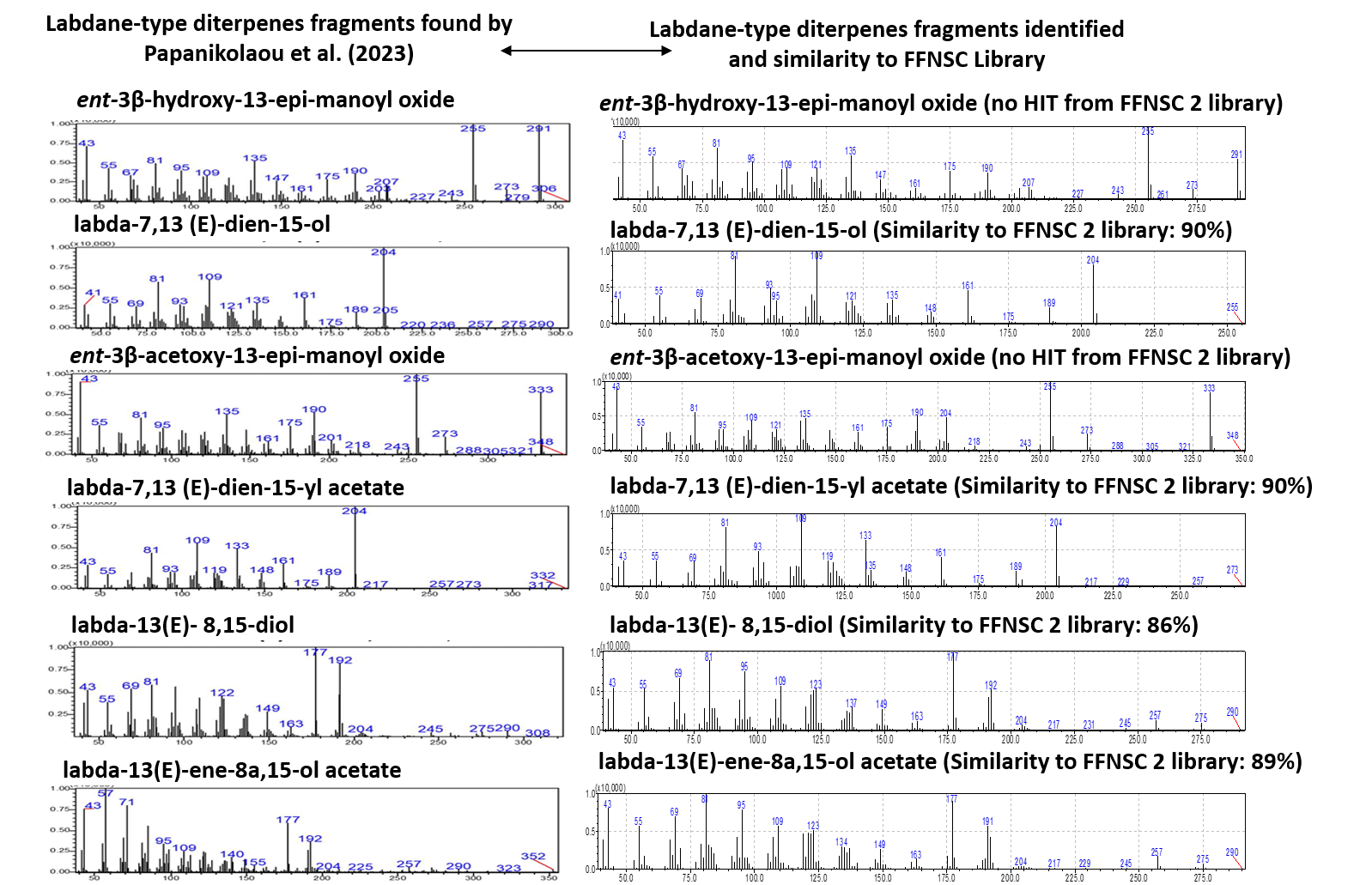


**Continued Fig. S2.** Mass spectra of the most abundant labdane-type diterpene peaks and their comparison with mass fragments from Papanikolaou et al. (2024) and the similarity identified with the FFNSC 2 GC-MS library.

**
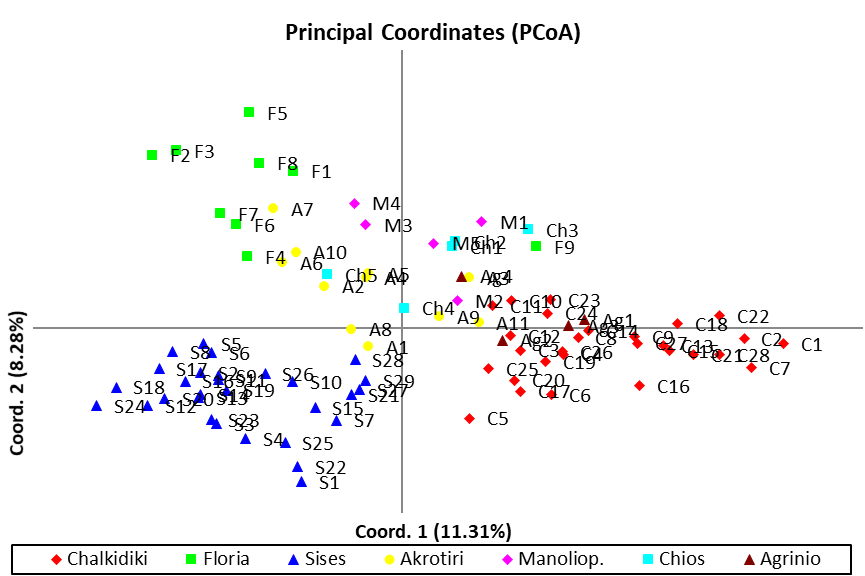
**

**Fig. S3** Principal Coordinates Analysis (PCoA) derived from bands amplified by 11 ISSR primers among the 7 populations and the 91 genotypes of *C. creticus* from Greece. Populations are distinguished by colored symbols, while genotypes by the letters next to each symbol. The *C. creticus* individuals grouped by PCoA as genotypes from Sises, in the blue circle, genotypes of Chalkidiki, in the red circle, and the genotypes from the other locations, in green circle.


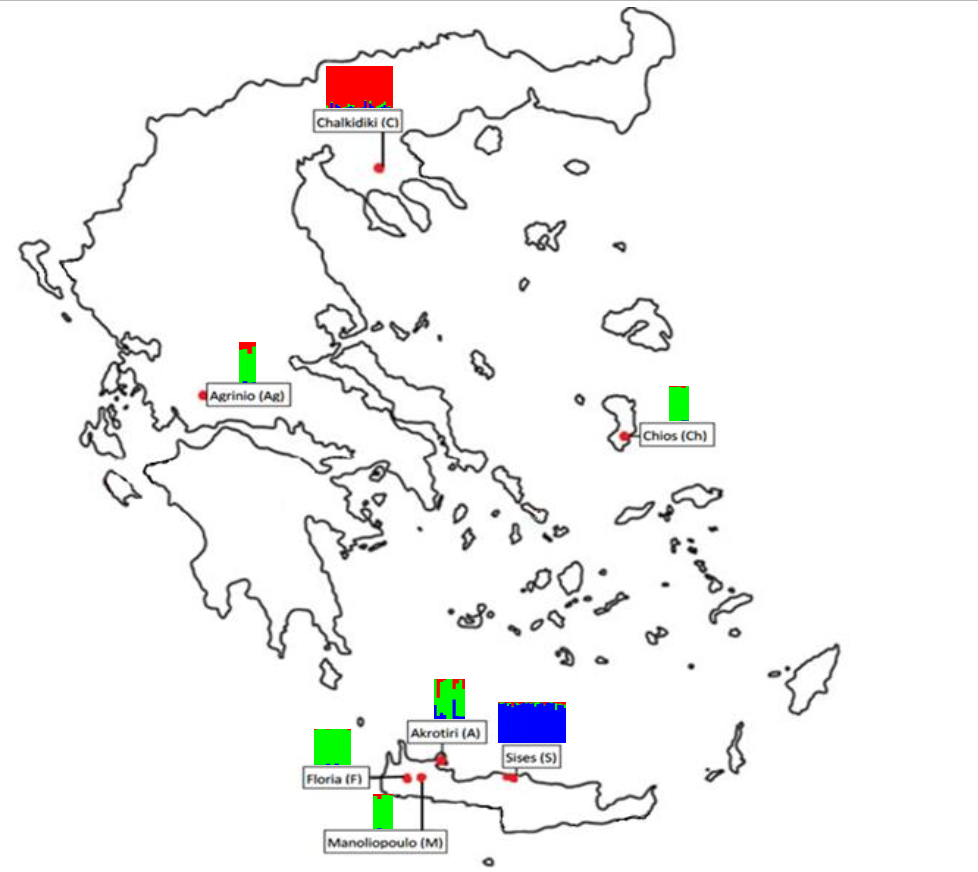
**Fig. S4** Geographical distribution (map of Greece) of the seven populations of C. creticus and STRUCTURE-based genetic relationships of the corresponding genotypes. Each point represents a population location, and the colored markers charts above each point, indicate the proportion of each genotype assigned to the respective three (ΔK=3) STRUCTURE clusters


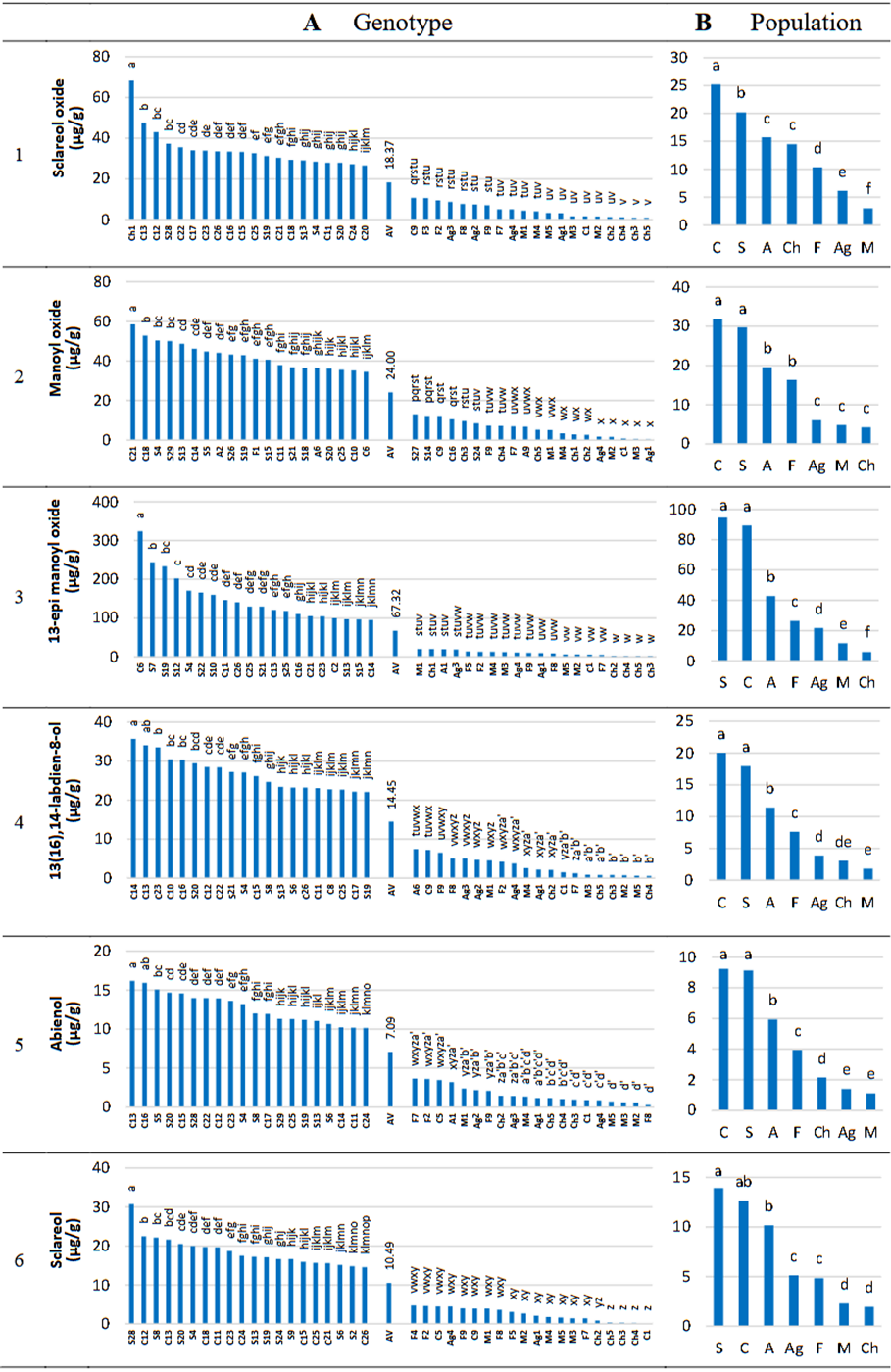


| 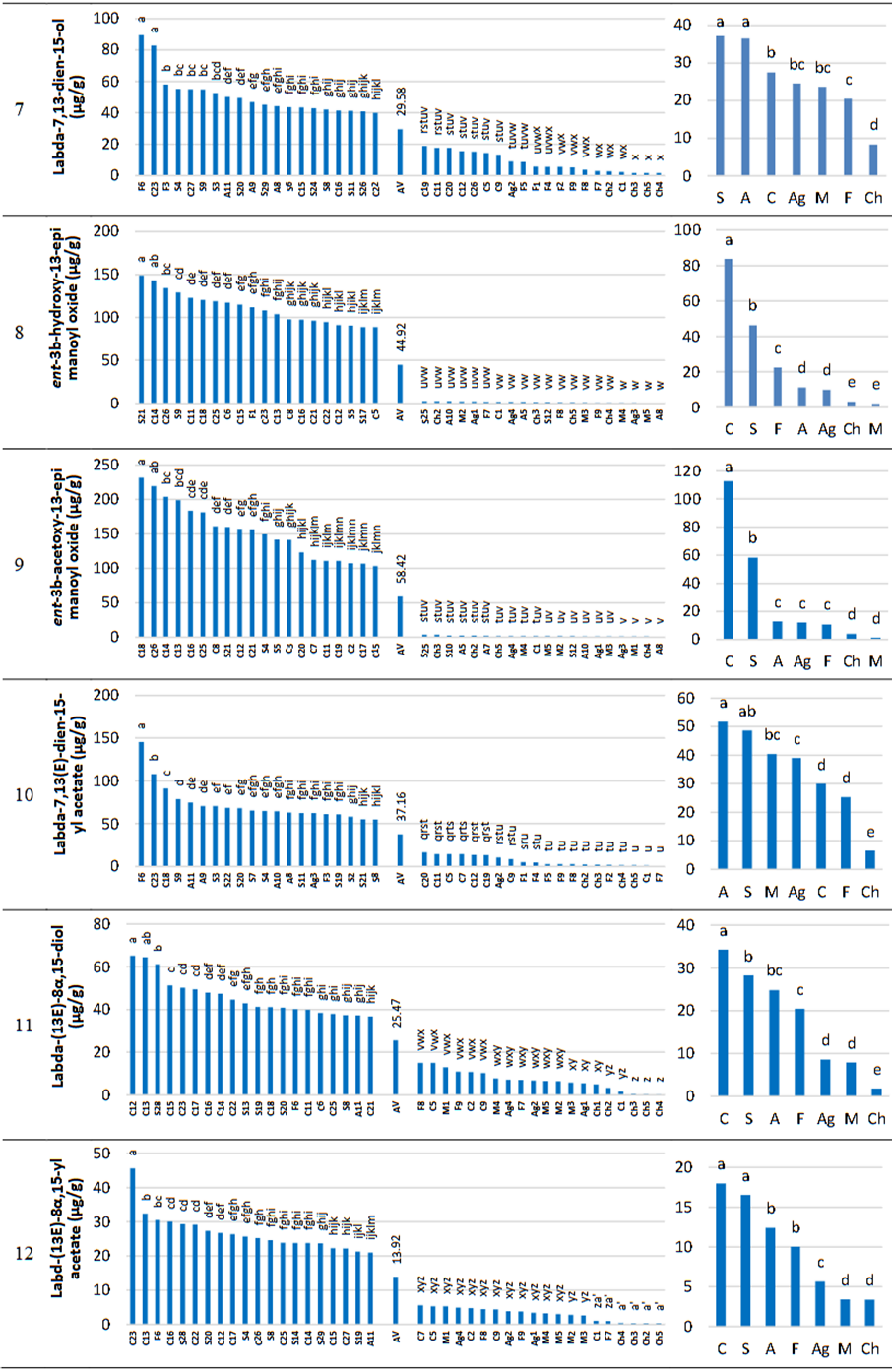  **Fig. S5** Concentrations of sclareol oxide, manoyl oxide, 13-epi-manoyl oxide, 13(16),14-labdien-8-ol, abienol, sclareol, labda-7,13-dien-15-ol, *ent-*3β-hydroxy-13-epi-manoyl oxide, *ent-*3b-acetoxy-13-epi-manoyl oxide, labda-7,13(E)-dien-15-yl acetate, labda-(13E)-8α,15-diol and labda-(13E)-8α,15-yl acetate in μg/g fresh young leaves of *C. creticus* growing in Experimental Field: (A) Allocation of the 91 genotypes (n=3, from sampling in three different plants-clones of the same genotype) of *C. creticus*. The figures present the 20 genotypes with the highest rates, the 20 genotypes with the lowest rates, and the average (AV) of all 91 genotypes. (B) The 7 populations (C: Chalkidiki, S: Sises, A: Akrotiri, F: Floria, Ch: Chios, Ag: Agrinio and M: Manoliopoulo) of *C. creticus* in descending order. Same letters indicate statistical similarity between rates (Duncan criterion, p <0.05)s |
| --- |


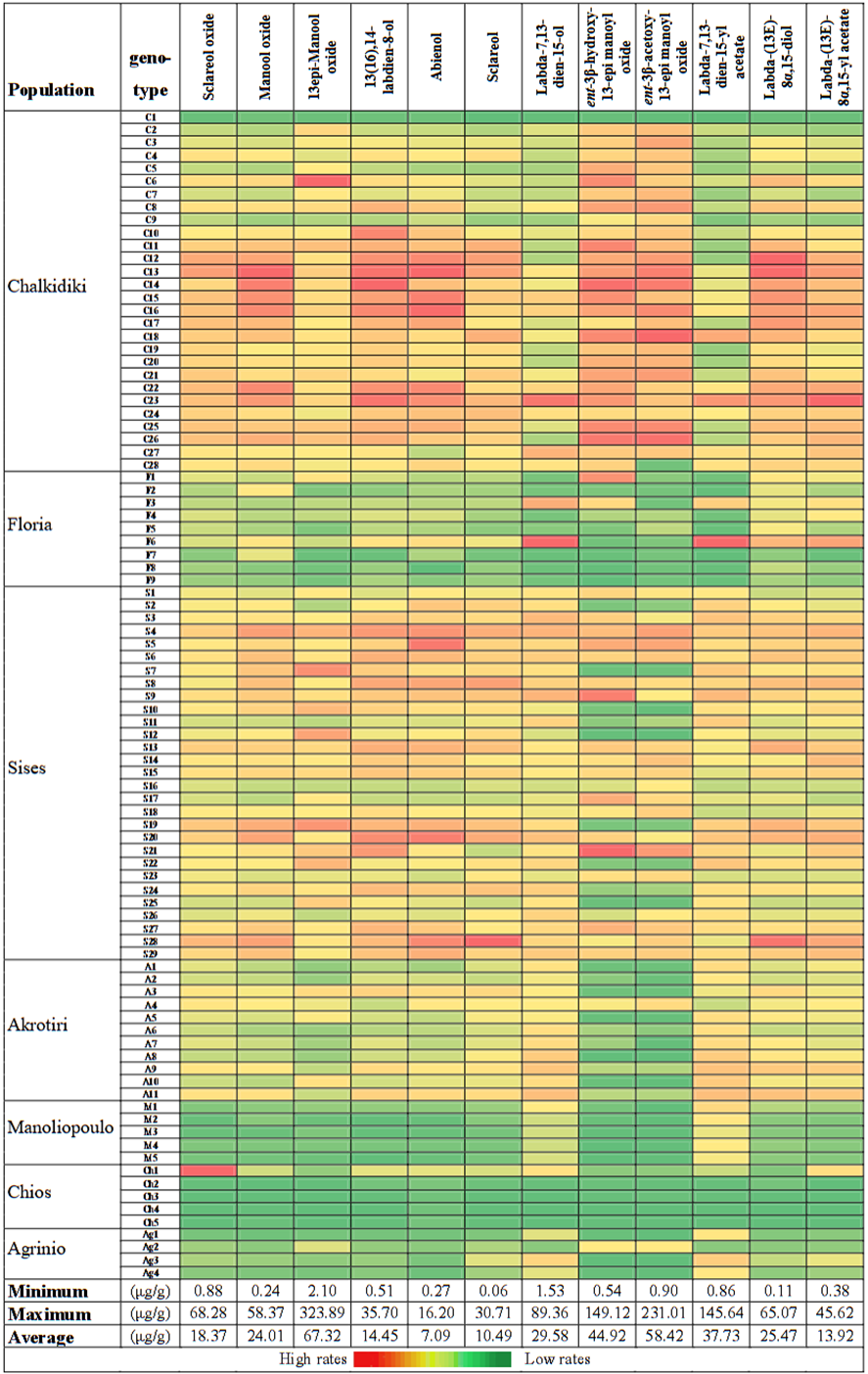


**Fig. S6** Heatmap of 12 labdane-type diterpenes detected in young leaves from 91 *C. creticus* genotypes versus their places of origin. Plants were growing in Aristotle University of Thessaloniki Experimental field (Thermi area), sampled from young leaves. Genotypes from Chalkidiki exhibited the highest values where those from Manoliopoulo and Agrinio had the lowest content.

| 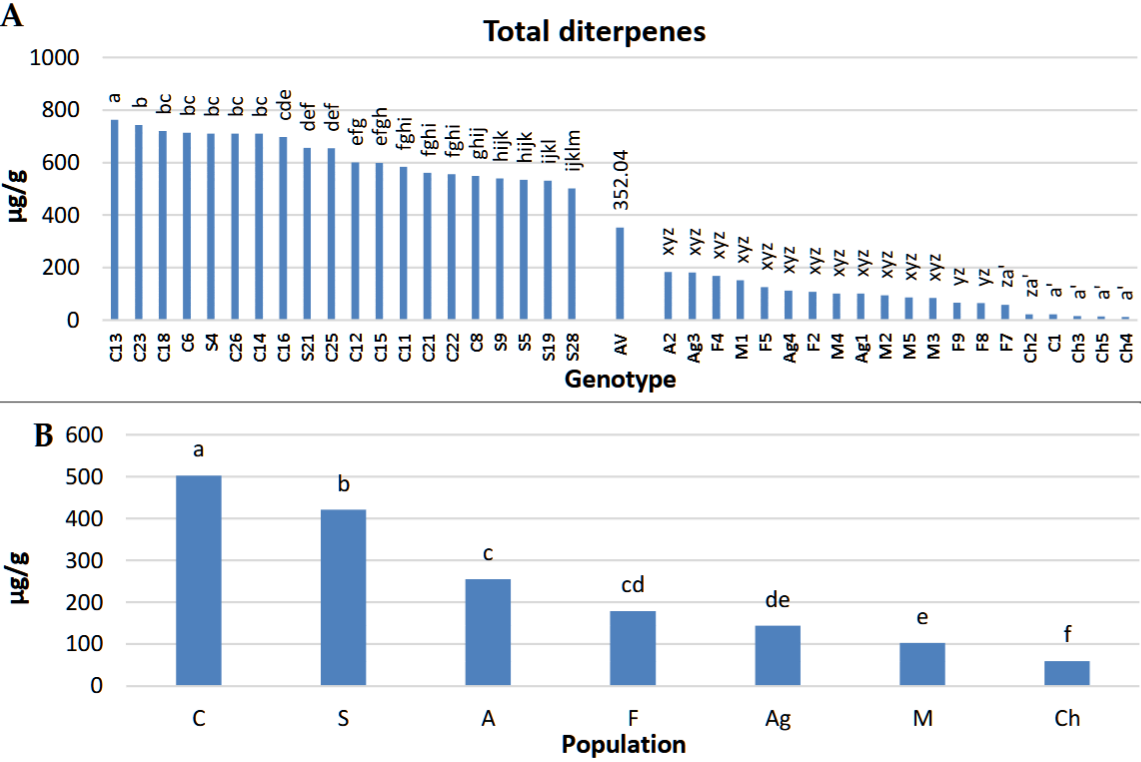 |
| --- |

**Fig. S7** Concentration of total labdane-type diterpenes in μg/g fresh young leaves of *C. creticus* growing in outdoor plant collection: (A) Allocation of the 91 genotypes (n=3, from sampling in three different plants-clones of the same genotype) of *C. creticus*. The main 40 genotypes are presented, the 20 with the highest rates, the 20 with the lowest rates and the average (AV) of all 91 genotypes. (B) The 9 populations of *C. creticus* in descending order. Codes indicate the origin of the genotypes C: Chalkidiki, S: Sises, A: Akrotiri, F: Floria, Ch: Chios, Ag: Agrinio and M: Manoliopoulo. Same letters indicate statistical similarity between rates (Duncan criterion, *p* <0.05).

| **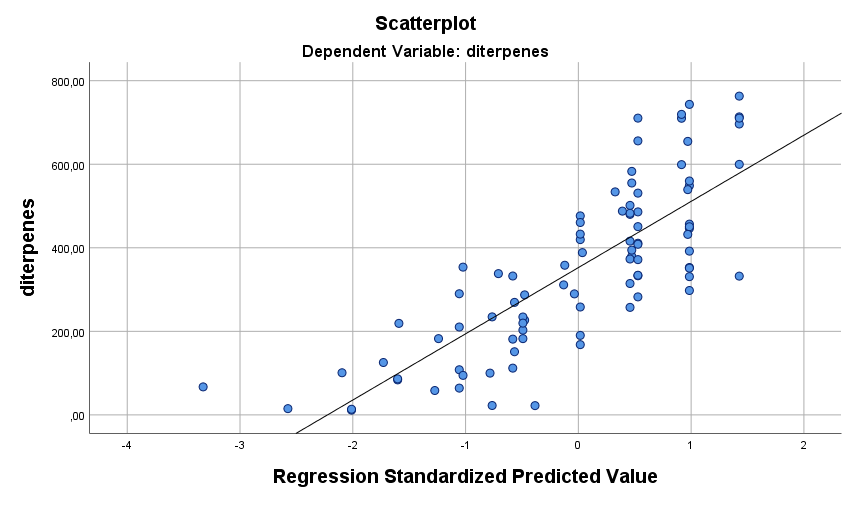** |
| --- |

**Fig. S8** Scatterplot showing the positive correlation of Regression Standardized Predicted Value and the total diterpenes production in *C. creticus* leaves, as determined by multiple regression analysis (MRA).

| 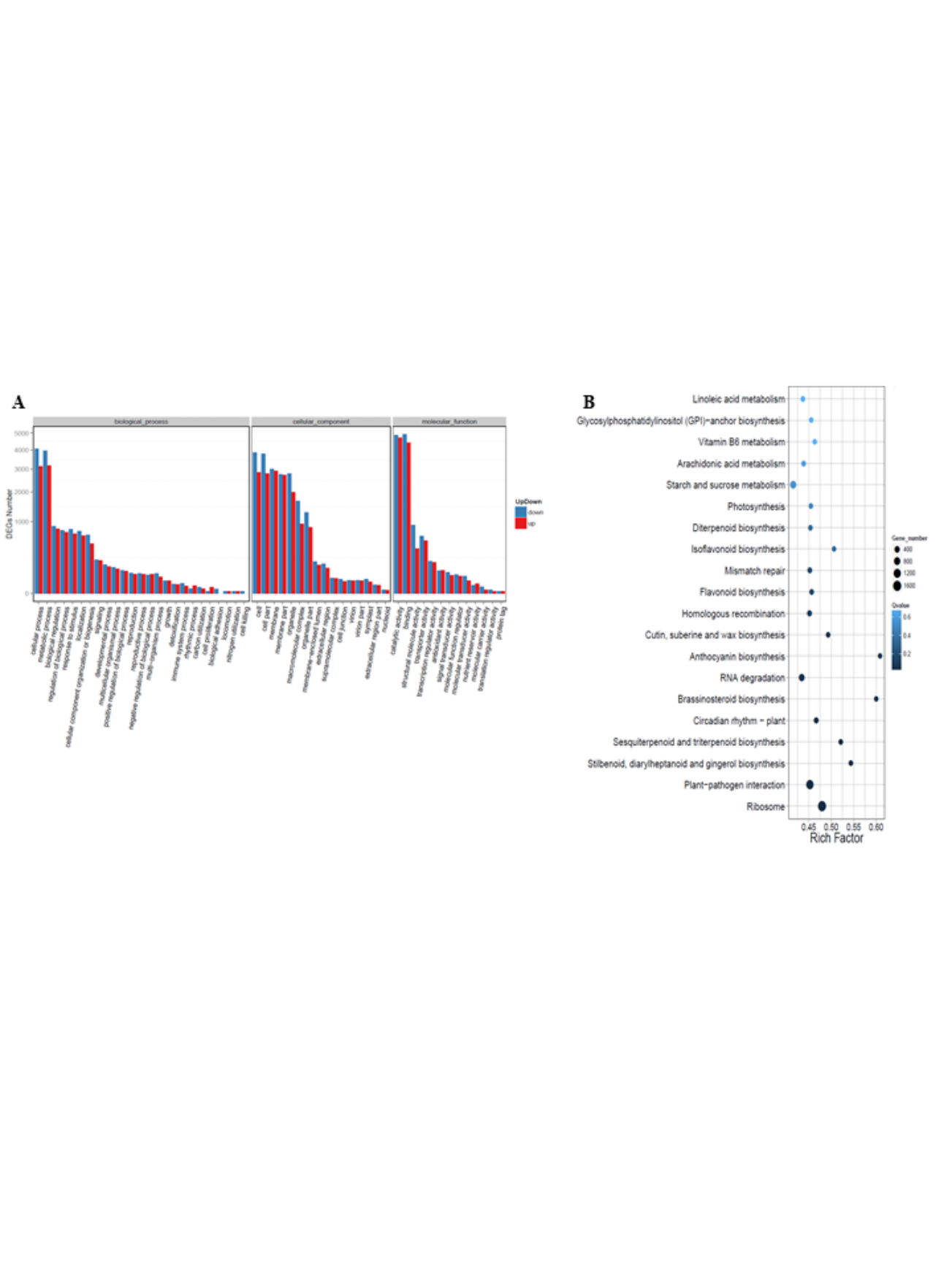 |
| --- |

**Fig. S9** A) Classification of the number of over- and under-expressed DEGs of C18 according to Gene Ontology. B) Functional enrichment of DEGs in the various biochemical pathways based on KEGG. The size of the circles represents the number of genes proportionally. The color refers to the q-value, (the lower is darker shade of blue), the more significant the enrichment. On the horizontal axis, the enrichment factor resulting from the quotient of the number of DEGs with the total number of genes, is another criterion of significance of functional enrichment. The higher its value, the more significant the enrichment.

| 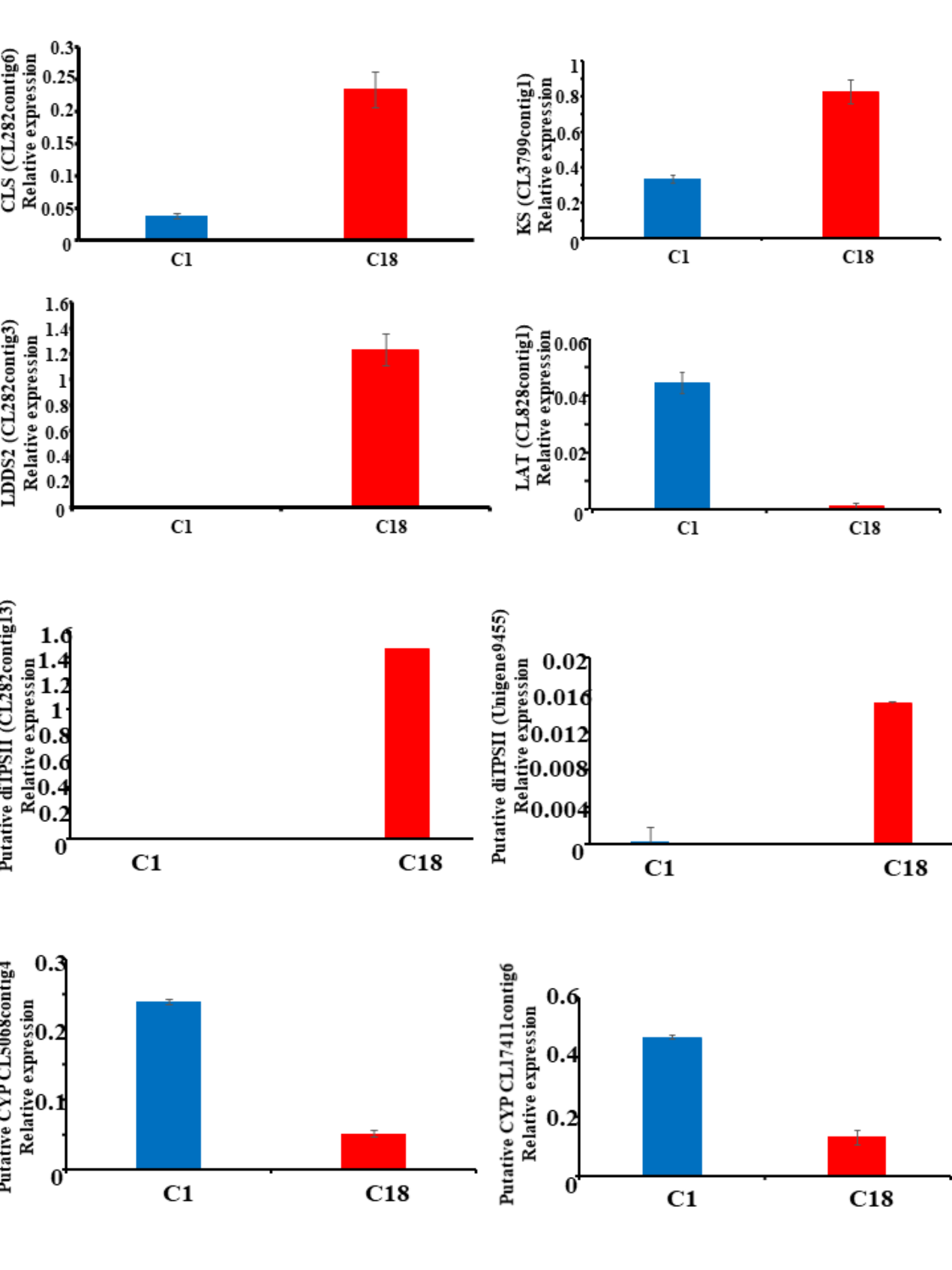 |
| --- |

**Fig. S10** Expression of key enzymes in the biosynthetic pathway of labdane diterpenes in leaves of *C. creticus*. Leaves of C1 (low) and C18 (high) genotypes that were used in this assay were at S1 developmental stage. *C. creticus* actin and elongation factor was used as reference genes, and expression of each gene was normalized to the average of *C. creticus* actin and elongation factor using the 2^-ΔCt^ method. Bars represent the mean of two biological replicates per genotype ± SE. Statistical significance was assessed with Student test at α=0.05. Abbreviations: *CLS: copal-8-ol diphosphate synthase; KS: kaurene synthase; LDDS2: labd-7,13(E)-dien-15-yl diphosphate; LAT: labdane acetyltransferase.*

| 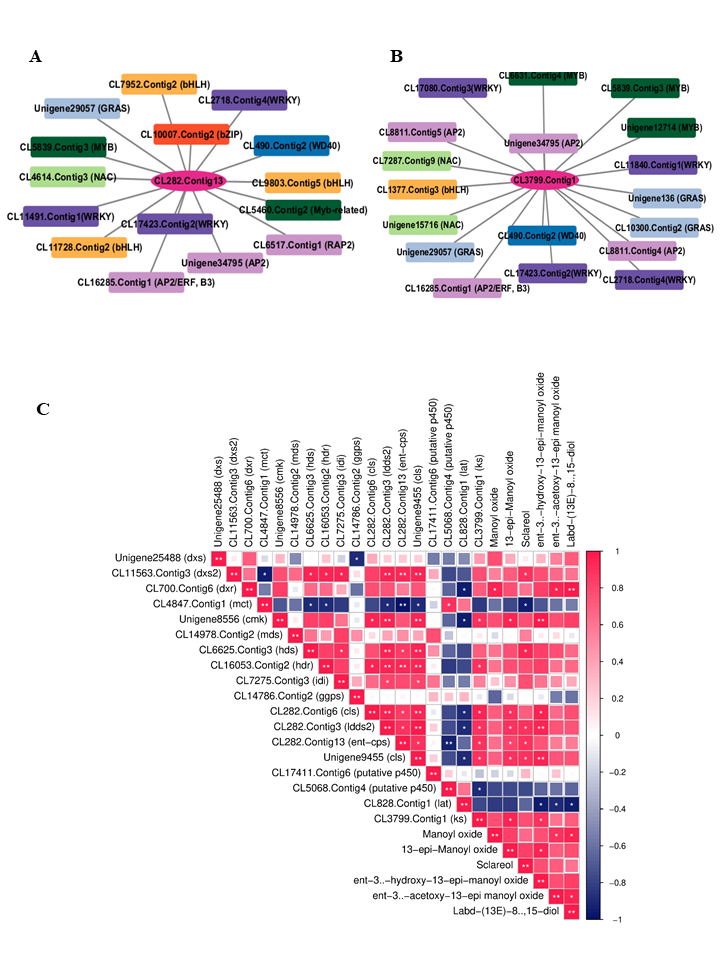 |
| --- |

**Fig. S11** Network analysis of Transcription Factors (rectangular shape) and genes (ellipse shape) of A) putative diTPSII *CL282contig13* and B) *kaurene synthase CL3799contig1* in the biosynthesis of the hydroxy-derivative of *ent*-13-epi-manoyl oxide, C) correlation analysis labdane diterpenes’ content and genes related to their biosynthesis. Significantly correlated TF–gene pairs (r≥ 0.95 and p <0.05) were selected to construct the transcriptional regulatory network.
